# Supplementary material for: Valence electron concentration as key parameter to control the fracture resistance of refractory high-entropy carbides
Source: Sci Adv. 2023 Sep 13;9(37):eadi2960. doi: 10.1126/sciadv.adi2960 (PMC10499311; doi:10.1126/sciadv.adi2960)
Supplement: 20230913-1 [file sciadv.adi2960.v1.pdf]

MATERIALS SCIENCE

Copyright © 2023 The Authors, some rights reserved; exclusive licensee American Association for the Advancement of Science. No claim to original U.S. Government Works. Distributed under a Creative Commons Attribution License 4.0 (CC BY).

Valence electron concentration as key parameter to control the fracture resistance of refractory high-entropy carbides

Davide G. Sangiovanni[1], Kevin Kaufmann[2]†, Kenneth Vecchio[2]*

Although high-entropy carbides (HECs) have hardness often superior to that of parent compounds, their brittleness—a problem shared with most ceramics—has severely limited their reliability. Refractory HECs in particular are attracting considerable interest due to their unique combination of mechanical and physical properties, tunable over a vast compositional space. Here, combining statistics of crack formation in bulk specimens subject to mild, moderate, and severe nanoindentation loading with ab initio molecular dynamics simulations of alloys under tension, we show that the resistance to fracture of cubic-B1 HECs correlates with their valence electron concentration (VEC). Electronic structure analyses show that VEC $\gtrsim$ 9.4 electrons per formula unit enhances alloy fracture resistance due to a facile rehybridization of electronic metallic states, which activates transformation plasticity at the yield point. Our work demonstrates a reliable strategy for computationally guided and rule-based (i.e., VEC) engineering of deformation mechanisms in high entropy, solid solution, and doped ceramics.

INTRODUCTION

Outstanding properties such as extremely high melting points, high hardness, and good resistance to erosion, corrosion, oxidation, and wear have driven ceramic applications toward technological uses, including protective coatings on tools and engine components, thin films for biomedical implants, space shuttle shielding tiles, and nuclear fuel pellets. Unfortunately, high hardness and high thermal stability—a manifestation of strong, stiff chemical bonds—are typically accompanied by brittleness, a problem of enormous technological concern. The ability to mitigate fracture could enable next-generation structural applications of ceramics such as hypersonic vehicle leading edges or nuclear reactors.

The recent advent of high-entropy ceramics—a class of alloys in which four, or more, metal elements, each present in concentrations above five atomic percent, are bonded to carbon, nitrogen, oxygen, boron, or silicon—opened possibilities for tuning or further enhancing mechanical and physical properties of ceramics, thus broadening the range of potential uses (*1*–*4*). Furthermore, high-entropy engineering of ceramics has greatly increased the range of possible ceramic compositions and therefore tunability, as it has been demonstrated that nonintuitive combinations will form a single phase (*5*–*8*). Among different families of multicomponent ceramics, rocksalt structure (B1) high-entropy carbide (HEC) alloys are suitable for applications that require bulk materials with ultrahigh melting temperatures (>3000 K) and mechanical strength. The hardness of the multicomponent material systematically surpasses values predicted by the rule of mixtures of binary constituents (*9*). However, the inherently low dislocation mobility of hard materials limits the possibility to dissipate mechanical stress via slip-induced plasticity, especially at room temperature. Static or cyclic loads may cause crack formation and/or propagation leading to sudden brittle failure. It is therefore imperative to identify rationales that allow enhancing fracture resistance (toughness) along with the material hardness.

Traditionally, trends in the ductility of solid crystals are predicted by phenomenological criteria based on elastic constants and moduli. For example, an approach often seen in the literature is to calculate the alloy Cauchy pressure (CP = $C_{12} - C_{44}$) or shear-to-bulk moduli ratio (G/B). Large positive Cauchy pressures (*10*) and G/B < 0.5 (*11*) indicate good deformability or compliance to shearing (reduced G and C_{44}).

Ab initio calculations of CP and G/B have been used to identify B1 structure carbides and nitrides with potentially improved ductility (*12*, *13*). In these ceramics, the shear stiffness is found to progressively decrease with increasing valence electron concentration (VEC). A relatively high VEC allows electrons to fully occupy bonding shear-sensitive metallic states near the Fermi level (*13*–*16*) and is expected to facilitate slip on {111} planes due to lowered stacking fault energies (*17*, *18*). Experimental testing has demonstrated superior fracture resistance in a single-crystal B1 nitride ($V_{0.5}Mo_{0.5}N$) with high VEC, large CP, and low G/B (*19*). In the case of $V_{0.5}Mo_{0.5}N$, enhanced ductility and toughness are attributed to dislocation plasticity, which produces substantial material pile-up during nanoindentation [figure 3 of (*19*)]. Although trends in CP and G/B appear to correlate with trends in ductility, predictions based on Pettifor and Pugh's criteria may overlook alloys in which an improved toughness originates from transformation-induced, not slip-induced, plasticity. For example, B1 nitride solid solutions or superlattices that contain AlN exhibit good toughness due to stress-activated B1 → B4 or B1 → B3 phase transitions within AlN-rich domains (*20*–*24*). In these ceramics, however, the calculated CP values are largely negative, as shown in table 2 of (*25*). Furthermore, a comprehensive atomistic understanding of intrinsic plastic behavior should not be confined to analyses of elastic responses, as implied in Pugh and Pettifor's criteria.

Based on experimental results and ab initio simulations of HECs subject to tensile deformation up to fracture points, we have recently proposed that the alloy VEC can be used to tune the alloy plasticity

[1]Department of Physics, Chemistry, and Biology (IFM), Linköping University, SE-581 83 Linköping, Sweden. [2]Department of NanoEngineering, UC San Diego, La Jolla, CA 92093, USA.
*Corresponding author. Email: kvecchio@ucsd.edu
†Present address: Oerlikon, San Diego, CA 92127, USA.

(*26*). Moreover, we have suggested that improved plasticity translates into increased materials resistance to brittle fracture. Nonetheless, it remains debated whether the experimental observations were incidentally consistent with the theoretical predictions given that only two samples with markedly different VEC have been considered in (*26*).

In the present work, we investigate six HEC compositions with VEC ranging from 8.4 to 9.6 e^-/f.u. to elucidate the correlation between the alloy VEC and its resistance to brittle fracture. We observe computationally and experimentally that HECs with VEC $\gtrsim 9.4$ e^-/f.u. exhibit markedly improved deformation responses, whereas alloys with VEC $\lesssim 9$ e^-/f.u. appear to be brittle. More unexpectedly, the computational studies suggest that the performance improvement results from transformation-induced plasticity by virtue of the ability to redistribute electrons into d-d bonds between next-nearest-neighbor metal atoms.

RESULTS AND DISCUSSION

We use ab initio molecular dynamics (AIMD) simulations accounting for the electronic spin degrees of freedom, bulk synthesis, and nanoindentation mechanical testing experiments to assess the plastic behavior and resistance to fracture of six HEC compositions. Simulations and experiments are performed for rocksalt structure (B1) HEC compositions of VEC between 8.4 and 9.6 e^-/f.u. (below, we omit the units "e^-/f.u." next to VEC values). The studied HEC compositions are listed in Table 1. The table also shows the alloys lattice parameters obtained by AIMD simulations at 300 K and experiments. Five alloys are investigated via both AIMD and experiments. For practical reasons, B1 (Ti,V,Cr,Mo,W)C is studied only via AIMD, whereas (V,Nb,Cr,Mo,W)C is investigated only experimentally.

The (001) surface is the easiest fracture plane for most B1 structure ceramics (*27*) due to its lowest formation energy $E_{surf}^{\{001\}}$. Present density functional theory (DFT) calculations verify that the relationship $E_{surf}^{\{001\}} < E_{surf}^{\{110\}} < E_{surf}^{\{111\}}$ holds for low-index HEC surfaces (see table S1). Thus, we primarily focus our discussion on results obtained by AIMD tensile testing along [001]. Figure 1 illustrates the tensile stress versus [001] elongation curves

Table 1. Sample chemistry, VEC, and structure information. List of investigated HEC compositions with corresponding lattice parameters *a* determined by AIMD at room temperature and experiments. Compositions marked in bold are here studied both in experiments and AIMD simulations.

HEC composition	VEC (e^-/f.u.)	*a* (Å)	
		AIMD	Experiments
▲ **(Ti,Zr,Hf,Nb,Ta)C**	8.4	4.507	4.52
■ **(Ti,Zr,Hf,Ta,W)C**	8.6	4.477	4.49
◆ **(Ti,V,Nb,Ta,W)C**	9.0	4.358	4.34
⬟ **(V,Nb,Ta,Mo,W)C**	9.4	4.370	4.35
⬢ (Ti,V,Cr,Mo,W)C	9.4	4.260	–
● **(V,Ta,Cr,Mo,W)C**	9.6	4.297	4.28
✶ (V,Nb,Cr,Mo,W)C	9.6	–	4.27

for the six investigated HEC compositions. We extract the room-temperature C_{11} and C_{12} elastic constants of the investigated alloys by polynomial fitting of the slope of σ_{xx}, σ_{yy}, and σ_{zz} stresses versus strain, for small [001] elongation. Note that Fig. 1 plots only the vertical σ_{zz} stress. Additional AIMD simulations are carried out to calculate the C_{44} from elastic shear stress responses during $\{001\}\langle 1\bar{1}0\rangle$ shearing. The method used for calculating elastic constants from stress/strain relationships has been detailed in (*28*). The bulk moduli of the alloys are obtained as $B = (C_{11} + 2\ C_{12})/3$, while the shear (G) and elastic (E) moduli are based on the Voigt-Reuss-Hill combination of elastic constants (*29*). The AIMD results are listed in Table 2 together with the shear (G) and elastic (E) moduli measured by sound wave velocities in the HEC samples. Overall, the experimental and ab initio results are in very good agreement (Table 2). Small discrepancies should be expected because of the slight carbon deficiency (typically around 10%) of the bulk synthesized materials (*9*, *30*, *31*). As shown in fig. S1, Cauchy pressures and G/B ratios vary linearly with the VEC. The monotonic increase in CP, and decrease in G/B, observed in fig. S1 is expected because of VEC-induced reduction in shear stiffness (Table 2). An increased occupation of metallic states near the Fermi level enhances constructive d-d wave interference during shearing [see schematic illustration in figure 8 (c and d) of (*13*)], thus counterbalancing the energetic penalty caused by deformation. Figure S1 suggests a continuous improvement in alloy ductility (and presumably toughness) as a function of the VEC, which is partially consistent with the experimental results discussed below.

AIMD stress/strain curves in Fig. 1 show that all HECs initially present a stiff response to [001] elongation, which reflects the large C_{11} values reported in Table 2. HECs with VEC = 8.4, 8.6, and 9.0 withstand tensile stress up to a maximum (theoretical strength) $\sigma_T^{[001]} \approx 26$ to 27 GPa. Subsequently, these alloys fracture in a brittle manner, which is indicated by the sudden drop in stress at elongations of 10 to 14% (filled symbols in Fig. 1). An example of HEC brittle cleavage is provided for the case of (Ti,Zr,Hf,Nb,Ta)C by the pink-framed simulation snapshot (inset in Fig. 1).

The HECs studied here with VEC = 9.4 or 9.6 exhibit slightly lower theoretical strength ($\sigma_T^{[001]} \approx 23$ to 25 GPa) than alloys with VEC ≤ 9.0. However, in contrast to the brittle alloys, HECs with VEC = 9.4 or 9.6 do not fracture but yield at strains of ≈8% via transformation-induced plasticity. A representative illustration is offered by the green-framed inset of Fig. 1, which shows that a large portion of the formerly cubic B1 structure of (V,Ta,Cr,Mo,W)C has transformed considerably due to local modifications in bond angles and atomic coordination. Strain-activated lattice transformations in alloys with VEC ≥ 9.4 effectively dissipate accumulated stress and delay rupture ("slow" bond fraying) up to [001] elongations of 22, 28, and 30%. Movies of HECs subject to [001] uniaxial strain can be found in the Supplemental Materials.

The correlation between mechanical response to uniaxial strain and VEC is further verified by AIMD simulations of HECs subject to [110] elongation (fig. S2), that is, normal to the surface of the second highest stability (table S1). Analogous to the results of [001] tensile testing, alloys with VEC < 9 cleave before yielding also during [110] elongation, whereas HECs with VEC ≥ 9.4 undergo structural modifications that substantially retard fracture (see fig. S2). To facilitate descriptions, here, we often refer to alloys with low (≤ 9.0) and high (≥ 9.4) VEC due to their statistically

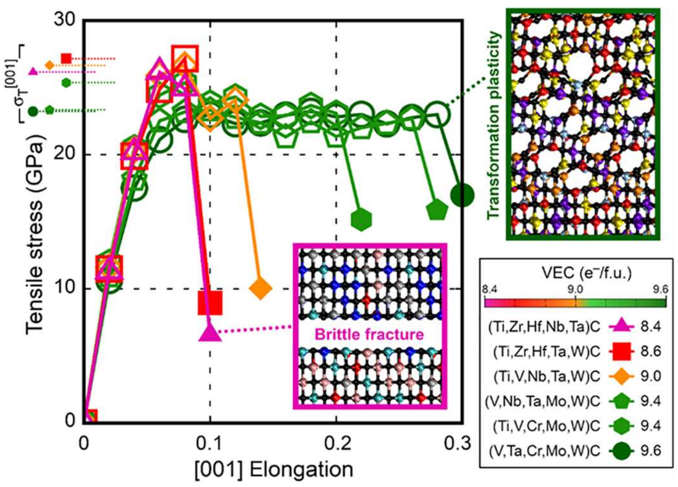

Fig. 1. AIMD tensile properties and deformation mechanisms. AIMD-calculated tensile stress $\sigma^{[001]}$ versus [001] elongation of B1 structure HEC supercells with different compositions and VEC. The filled symbols indicate fracture points. Small filled symbols on the left indicate tensile strengths $\sigma_T^{[001]}$ values. The insets illustrate AIMD snapshots of brittle fracture in (Ti,Zr,Hf,Nb,Ta)C (10% elongation) and strain-induced transformation plasticity in (V,Ta,Cr,Mo,W)C (28% elongation). Chemical bonds have a maximum length of 2.6 Å. Movies can be found in the Supplementary Materials. Sphere color legend: black = C, silver = Ti, cyan = Zr, blue = Hf, ice-blue = V, pink = Nb, red = Ta, violet = Cr, yellow = Mo, orange = W.

significant difference in fracture resistance. Table 1 should be consulted to find correspondence between alloy compositions and VEC.

Although strain is along the [110] crystal axis, the fractured surface of low-VEC HECs exposes {001} facets (pink-framed inset in fig. S2). This is due to the propensity of brittle ceramics to break along the easiest cleavage planes. Crack paths observed during [111] uniaxial strain of other B1 structure ceramics also follow lowest-energy {001} surfaces [see the case of TiN in figure 10 of (*22*)]. Conversely, for high-VEC HECs, [110] elongation beyond the yield point progressively induces B1 → graphitic-like lattice transformations. The transition entails a change in atomic coordination from sixfold to fivefold, where each metal/carbon is bonded to three carbons/metals of the same hexagonal layer and two carbons/metals of adjacent layers (green-framed inset in fig. S2). Owing to transformation plasticity, HECs with VEC ≥ 9.4 withstand an elongation of 20% or higher. Rupture occurs by relatively slow bond fraying of honeycomb-patterned domains.

It should be emphasized that the extremely high stress and elongation recorded during AIMD simulations (Fig. 1 and fig. S2) represent ideal upper bounds to actual properties attainable in an HEC. For example, the absence of native defects (such as dislocations and grain boundaries) and very high strain rates used in atomistic simulations return values of strength and elongation at fracture that are much larger than in most experiments. The experimental compressive strength of submicrometer monolithic binary carbides reaches ≈20 GPa (*32*, *33*). Such high values (accidentally close to AIMD theoretical strengths σ_T; table S2) are due to the high ceramic resistance to compression and low density of native defects in submicrometer single-crystal samples. Fracture strengths obtained for polycrystalline B1-structured nitrides by microcantilever bending are ≈4 to 6 GPa (*34*), nearly 10 times smaller than σ_T evaluated by AIMD (table S2). Fracture of the cantilever, however, is likely to initiate at grain boundaries. During nanoindentation, nanometer-scale HEC domains around indenter corners may plausibly withstand local strains of a few percent before crack initiation. In HEC bulk ceramics under load, strain-mediated lattice transformations are expected to operate at nanometer length scales. Despite limitations inherent to atomistic simulations, transformation plasticity mechanisms predicted by AIMD may realistically portray small-scale yielding, thus explaining the enhanced fracture resistance of HECs with VEC ≳ 9.4 (see experimental results below).

As described in the next paragraph, we carried out a series of nanoindentation tests (details given in Materials and Methods) to gain statistical information on the fracture resistance of the synthesized HEC samples listed in Table 2. Experimental characterization of our polycrystalline samples verifies that trends in fracture resistance versus VEC are not qualitatively affected by the presence of secondary phases nor differences in preferred orientations of the crystallites. X-ray diffraction analysis (fig. S3) shows that only a single face-centered cubic phase (B1 structure) is present in our samples. Moreover, energy-dispersive x-ray spectroscopy measurements reveal that the experimental compositions match the targeted nominal compositions (table S3). The grains of our samples are crystallites with an average size of ≈25 to 40 μm, which is indicated by electron backscatter diffraction (EBSD) orientation maps in fig. S4. This lends confidence that our nanoindentation results reflect the mechanical properties inherent to the B1 structure solid solutions and that the statistics of fracture resistance should be negligibly biased by grain boundary properties. We also analyze the grain orientation of the synthesized HECs. The texture of the samples is measured using the multiples of a uniform distribution metric from the EBSD orientation maps and the inverse pole figures of fig. S5. The results demonstrate that the crystallites are essentially randomly oriented. Thus, the combination of the information collected by constant-load and binned-depth nanoindentation provides a comprehensive qualitative understanding of the fracture resistance of single-phase B1 structure HEC samples and its correlation with the alloy VEC.

The statistics of HEC fracture resistance as a function of nanoindenter load and penetration are summarized in Fig. 2. Additional details are given by histograms in fig. S6. As expected, the percentage of tests exhibiting fracture increases quasi-monotonically with increasing load (L) (Fig. 2A) or penetration (P) (Fig. 2B). However, tests performed on alloys with low VEC immediately exhibit a substantial percentage of cracks for the lowest applied load and smallest sample penetration. At a nanoindenter load of 50 mN and penetration depth of 200 nm, (Ti,Zr,Hf,Nb,Ta)C (purple triangles), (Ti,Zr,Hf,Ta,W)C (red squares), and (Ti,V,Nb,Ta,W)C (orange diamonds) fracture in ≈20 ± 10% of the total number of mechanical tests (Fig. 2, A and B). At the same experimental conditions, the high-VEC alloys (V,Nb,Ta,Mo,W)C and (V,Ta,Cr,Mo,W)C (green pentagons and circles) display surface cracks in very few cases (≈0 to 3%). The behavior of the alloy (V,Nb,Cr,Mo,W)C (VEC = 9.6; green stars in Fig. 2) is somewhat intermediate to that of HECs with low and high VEC. A separate discussion dedicated to this material composition follows in the next paragraph. As the nanoindenter load or penetration in low-VEC samples increases to L = 300 mN and P = 600 nm, the fraction of tests that exhibit cracks rapidly raises to nearly 100%. A further increase in load or penetration produces cracks in all tests performed, as indicated by the trends of triangle,

Table 2. Computational and experimental elastic properties of HECs. Cubic C_{11}, C_{12}, and C_{44} elastic constants, bulk (B), shear (G), and elastic (E) moduli of polycrystalline materials (Voigt-Reuss-Hill average used for theoretical results), G/B ratios, and Cauchy's pressures (CP = $C_{12} - C_{44}$) of HECs determined by AIMD and experiments. Measured elastic and shear moduli are given in parentheses. The AIMD elastic constants of (Ti,Zr,Hf,Ta,W)C and (V,Nb,Ta,Mo,W)C are taken from our previous work (*28*). AIMD-calculated elastic constants C_{11}, C_{12}, and C_{44} are extracted from the derivative of stress versus strain curves at vanishingly small strain, as described in (*28*). Compositions marked in bold are here studied both in experiments and AIMD simulations.

HEC composition	C_{11} (GPa)	C_{12} (GPa)	C_{44} (GPa)	B (GPa)	E (GPa)	G (GPa)	G/B (%)	CP (GPa)
▲ **(Ti,Zr,Hf,Nb,Ta)C**	**612**	**110**	**180**	**277**	**495 (460 ± 20)**	**206 (190 ± 10)**	**74**	**-70**
■ **(Ti,Zr,Hf,Ta,W)C**	**619**	**122**	**165**	**288**	**475 (465 ± 20)**	**194 (185 ± 10)**	**68**	**-43**
◆ **(Ti,V,Nb,Ta,W)C**	**630**	**148**	**170**	**309**	**484 (475 ± 20)**	**196 (195 ± 10)**	**63**	**-22**
⬟ **(V,Nb,Ta,Mo,W)C**	**616**	**159**	**145**	**311**	**440 (450 ± 20)**	**174 (180 ± 10)**	**56**	**14**
⬢ (Ti,V,Cr,Mo,W)C	633	150	138	311	438 (–)	173 (–)	56	12
● **(V,Ta,Cr,Mo,W)C**	**578**	**160**	**128**	**299**	**399 (420 ± 20)**	**156 (175 ± 10)**	**52**	**32**
✶ (V,Nb,Cr,Mo,W)C	–	–	–	–	–(405 ± 20)	–(170 ± 10)	–	–

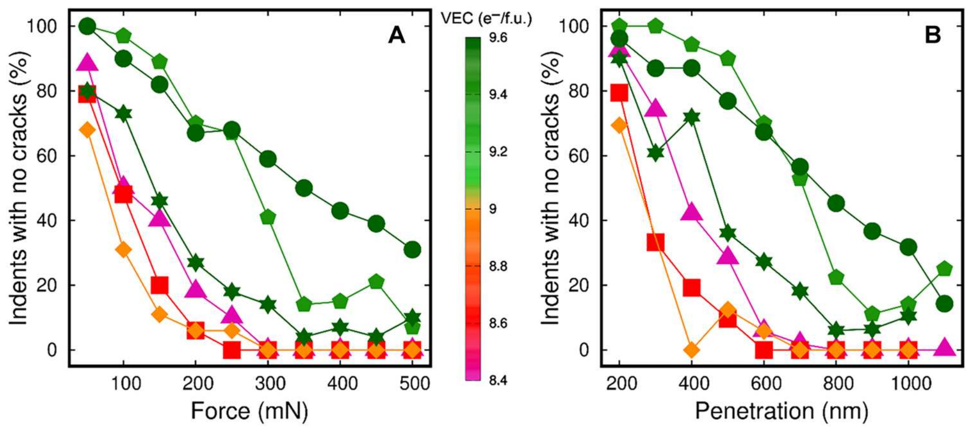

Fig. 2. Statistics of fracture resistance collected via nanoindentation. Mechanical testing at constant load (**A**) and binned penetration depth (**B**) [note missing data points for (Ti,Zr,Hf,Ta,W)C, (Ti,V,Nb,Ta,W)C, and (V,Nb,Cr,Mo,W)C at P = 1100 nm and for (Ti,V,Nb,Ta,W)C at P = 300 nm]. Detailed results of nanoindentation tests can be found in fig. S6. The figure shows alloys with VEC ≤ 9.0 fracture in 100% of the tests for applied forces exceeding 250 mN or for penetrations ≥800 nm.

square, and diamond symbols in Fig. 2 (A and B) for L ≥ 300 mN and P ≥ 600 nm. Conversely, for intermediate stress conditions (L = 300 mN and P = 600 nm) the high-VEC alloys (V,Nb,Ta,Mo,W)C and (V,Ta,Cr,Mo,W)C fracture in only 30 to 60% of the performed tests. These alloys maintain a substantial resistance to fracture up to the most severe conditions of stress that we have tested. This is seen in trends of green pentagons and circles for the ranges of load 300 < L ≤ 500 mN and penetration 600 < P ≤ 1100 nm in Fig. 2 (A and B).

As mentioned above, mechanical testing reveals that (V,Nb,Cr,Mo,W)C (green star symbols in Fig. 2) is less resistant to fracture than the other investigated high-VEC alloys [(V,Nb,Ta,Mo,W)C and (V,Ta,Cr,Mo,W)C]. Nevertheless, the statistics of Fig. 2 (A and B) show that the resistance to fracture of (V,Nb,Cr,Mo,W)C is superior to that of all low-VEC HECs. In general, the results of Fig. 2 evidence the effect of VEC on alloy fracture resistance. Under severe stress (L ≥ 300 mN) and deformation (P ≥ 800 nm) conditions, all low-VEC alloys fracture in 100% of the tests, while all high-VEC alloys resist fracture in a considerable number of cases: between a minimum of ≈5% and up to a maximum of ≈60%.

An alternative analysis of experimental results presented in Fig. 2 allows us to better appreciate the differences in alloy fracture resistance as a function of VEC. No matter how brittle, essentially any hard ceramic material is expected to resist fracture for a small depth or force applied by external bodies. Therefore, a metric of effective fracture resistance should account—besides the percentage of tests exhibiting no cracks—for the deformation and load induced by the nanoindenter. Figure 3 (B and C) proposes a metric (named effective fracture resistance) of effective HEC resistance to fracture during depth-controlled (I_{FR}^{P}) and force-controlled (I_{FR}^{L}) nanoindentation testing. In Fig. 3 (B and C), the percentage of nanoindentation tests in which an alloy does not crack $\left(\frac{N_{no\ cracks}}{N_{Tests}}\right)$ contributes to the fracture resistance as a linear factor of deformation

$$I_{FR}(P) = P \cdot \left(\frac{N_{no\ cracks}^{P}}{N_{Tests}^{P}}\right) \tag{1a}$$

and load

$$I_{FR}(L) = L \cdot \left(\frac{N_{no\ cracks}^{L}}{N_{Tests}^{L}}\right) \tag{1b}$$

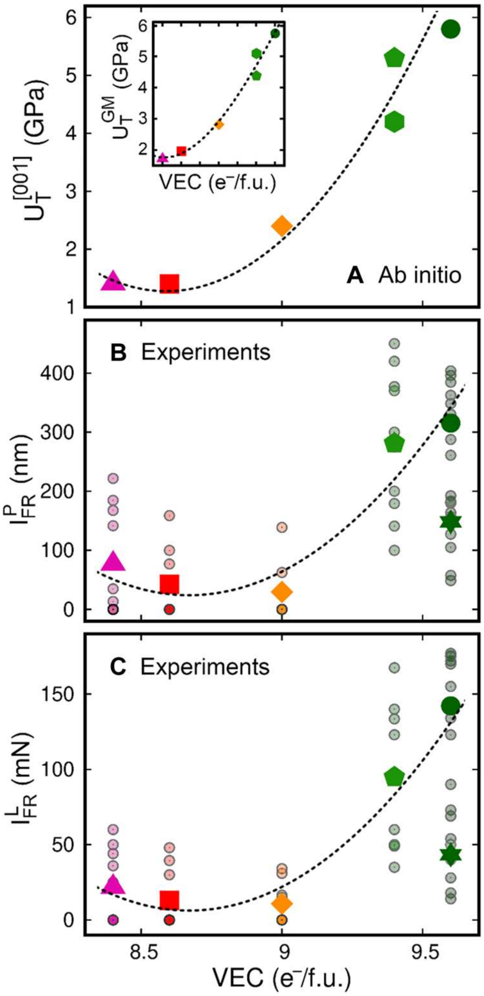

Fig. 3. Effective fracture resistance of the HECs. Comparison of trends in (**A**) ideal tensile toughness $U_T^{[001]}$ obtained by AIMD and (**B** and **C**) materials resistance to fracture (I_{FR} metrics described in the text) assessed by nanoindentation as a function of VEC. The inset in (A) shows the VEC dependence of $U_T^{GM} = (U_T^{[001]} \cdot U_T^{[110]})^{½}$, the geometric mean of tensile toughness recorded for strain along [001] and [110]. $U_T^{[001]}$ and $U_T^{[110]}$ values are listed in table S2. In (B) and (C), the small shaded-colored circles are the percentage of fracture-resistant tests weighted by (B) depth of the nanoindenter (Eq. 1a) and (C) load of the nanoindenter (Eq. 1b). The filled color symbols are effective fracture resistances $[(I_{FR}^{P})$ and $(I_{FR}^{L})]$ calculated for each material using arithmetic averages in Eq. 2a and Eq. 2b. The dashed lines serve as guides to follow trends in toughness U_T and fracture resistance obtained for the five HEC compositions (▲ ■ ◆ ⬟ ● symbols in Table 2) studied both in simulations and experiments.

induced by a nanoindenter. The labels L and P indicate results recorded at load L or penetration P. $I_{FR}(P)$ and $I_{FR}(L)$ values calculated for each penetration or load are indicated by small semitransparent colored circles in Fig. 3 (B and C, respectively). Some circles appear more intensely shaded than others owing to overlapping data points. Thus, the effective fracture resistance assessed for each material—indicated by large colored symbols in Fig. 3 (B and C)—is obtained as

$$I_{FR}^{P} = \sum \frac{I_{FR}(P)}{n_P} \tag{2a}$$

and

$$I_{FR}^{L} = \sum \frac{I_{FR}(L)}{n_L} \tag{2b}$$

In Eq. 2a and Eq. 2b, n_P and n_L are the number of different nanoindenter depths and loads tested on a given material. The trends of Fig. 3 (B and C) indicate that alloy fracture resistance (I_{FR}) correlates with its VEC.

The plots in Fig. 3 (B and C) also allow us to suggest a direct comparison between HEC fracture resistances assessed by experiments and AIMD simulations. Figure 3A summarizes the total tensile toughness $U_T^{[001]}$ evaluated via AIMD simulations of defect-free single-crystal alloy models strained along [001]. AIMD-calculated $U_T^{[001]}$ values (stress/strain areas in Fig. 1) set an ideal upper bound to the strain energy density that can be absorbed by an HEC alloy up to fracture. U_T is therefore chosen as an ab initio descriptor of intragrain fracture resistances of HECs subject to [001] elongation. Similar to experimental results in Fig. 3 (B and C), ab initio results in Fig. 3A show that alloy fracture resistance is enhanced significantly for VEC greater than 9.0. The similarity between experimental and AIMD trends appears even more clearly if one restricts the comparison to alloys investigated via both nanoindentation and simulations (follow dashed lines passing close to ▲ ■ ◆ ⬟ ● symbols in Fig. 3).

Although primary, the VEC and/or transformation-induced plasticity are not the only factors affecting HEC fracture resistance. For example, within a grain of ceramic, point defects, dislocations, and their interactions may also play beneficial or detrimental roles in the material plastic response (*35*, *36*). As inferred by calculated CP and G/B versus VEC trends (fig. S1), a higher electron concentration may facilitate lattice slip, besides enabling transformation toughening. Moreover, the choice of metal elements and the precise ratio in which they are mixed to achieve a target VEC will also play a role, as demonstrated by (V,Nb,Cr,Mo,W)C. Nevertheless, the trends presented in Fig. 3 point to a strong correlation between the mechanical behavior of defect-free single-crystal models and actual HEC subject to nanoindentation.

Effect of VEC on hardness, transformation plasticity, and fracture resistance

Nanosized cracks and structural flaws are inevitably present in manufactured bulk ceramics. High Peierls stresses (*36*, *37*) compared to metals limit the ability of ceramics to emit dislocations from crack tips (*38*). Accordingly, mechanically and thermally accumulated stresses may cause crack growth leading to brittle failure. However, plasticity mechanisms that are not mediated by dislocations may enhance toughness (fracture resistance) while preserving strength and hardness. For example, stress-activated lattice transformations—known to operate in ZrO_2 and ZrO_2-based ceramics (*39*, *40*)—create a plastic zone ahead of crack tips, which arrests crack propagation and enhances the material resistance to fracture.

Static and cyclic tensile (mode I) stresses are the primary cause of service failure in ceramics. During nanoindentation, penetration of the tip induces severe tensile stresses around the indenter corners, where radial (Palmqvist) cracks are initiated (*41*). However, the strain-activated lattice transformation predicted by AIMD for high-VEC HECs (see insets of Fig. 1, fig. S2, and movies S3 and S4) suggests that these materials may behave similarly to ZrO_2

when subject to mode I loading. Nanometer-scale lattice transformations may delay or even prevent the crack formation and/or propagation by creating plastic zones ahead of the crack, thus explaining the good resistance to fracture exhibited by high-VEC HECs in Figs. 2 and 3. Here, we should emphasize that the focus of our study is to rank HECs according to relative differences in fracture resistance, not to quantify their mode I fracture toughness K_{Ic}. Among ceramics, a high fracture toughness ($K_{Ic} \approx 5$ to 15 MPa $m^{½}$) often comes at the expense of low hardness (H ≈ 5 to 10 GPa), as seen for example in ZrO_2-based alloys (*42*–*45*) and zeta-phase carbides (*46*). HECs are, instead, very hard ceramics [H ≈ 25 to 35 GPa (*9*)] and the expected K_{Ic} values correspondingly lower, making valid measurements all the more difficult. An accurate characterization of the inherent intragrain fracture toughness of these alloys is not feasible, as it would require the preparation and testing of large monolithic B1 samples. Expedients often adopted—that is, measuring the length of cracks produced by indenting individual grains—are quantitatively unreliable (*47*, *48*).

Although nanoindentation testing and AIMD simulations show that high-VEC HECs exhibit superior fracture resistance and the ability to transform plastically, their experimentally measured hardness (H) and theoretical strength ($\sigma_T^{[001]}$) are comparable to that of low-VEC alloys. The values recorded for the investigated HECs are all within the ranges 27 ≤ H ≤ 33 GPa and 23 ≤ $\sigma_T^{[001]}$ ≤ 27 GPa (Fig. 4). In contrast, the $\sigma_T^{[110]}$ theoretical strength decreases (from 38 to 26 GPa) with increasing VEC more rapidly than $\sigma_T^{[001]}$. AIMD results of theoretical strength $\sigma_T^{[001]}$ and $\sigma_T^{[110]}$, toughness $U_T^{[001]}$ and $U_T^{[110]}$, and elongation at fracture $\delta_f^{[001]}$ and $\delta_f^{[110]}$ are summarized in table S2.

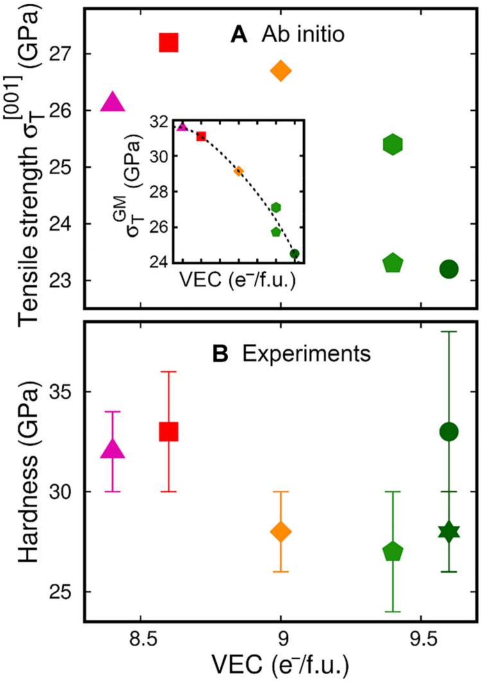

Fig. 4. Comparison of ideal tensile strength and nanoindentation hardness. AIMD-calculated ideal tensile strength $\sigma_T^{[001]}$ (**A**) and nanoindentation hardness (**B**) plotted as a function of the alloy VEC. The inset in (A) shows the VEC dependence of $\sigma_T^{GM} = (\sigma_T^{[001]} \cdot \sigma_T^{[110]})^{½}$, the geometric mean of tensile strength recorded for strain along [001] and [110]. $\sigma_T^{[001]}$ and $\sigma_T^{[110]}$ values are listed in table S2.

Tailoring the intrinsic properties of B1 single-crystal carbonitrides by controlling the VEC has been reported previously. Based on an analysis of electronic structures and a rigid-band-filling model, Jhi *et al.* (*15*) showed that the hardness of transition metal (TM) carbonitride alloys exhibits maximum values for VEC around 8.4 e^-/f.u. because of full occupation of shear-resistant p-d nonmetal/metal bonds combined with a negligible population of shear-sensitive d metallic states close to the Fermi level. This is supported, for example, by trends in microhardness versus VEC of B1 structure ceramics [see table 1 in (*49*)]. In analogy to Jhi's theory, Fig. 4B shows that the trend in nanoindentation hardness of HECs has a maximum for VEC near 8.6 e^-/f.u. An exception is the hardness measured for (V,Ta,Cr,Mo,W)C (green filled circle in Fig. 4B), which deviates from the theoretically predicted trend. As will be discussed in greater detail in the section "Deviations of mechanical properties from theoretical trends and future research directions," discrepancies between theoretical and actual trends in mechanical properties are expected. Similar to hardness, the theoretical tensile strength σ_T reaches its maximum for VEC near 8.5 e^-/f.u. (Fig. 4A and its inset). Hardness and strength are often correlated in solids (*50*–*52*). Opposite to trends in strength, the theoretical toughness U_T of HECs increases superlinearly for VEC higher than ≈8.5 (Fig. 3A and its inset). Thus, the results of Figs. 3 and 4 suggest that trends in theoretical strength and toughness—promptly obtainable via AIMD simulations—may be used to forecast trends in hardness and fracture resistance of B1 structure carbides and nitrides.

High hardness and strength (Fig. 4), combined with enhanced resistance to fracture (Figs. 2 and 3), indicate high-VEC HECs as promising ceramic material candidates for structural and engineering applications that require high hardness, heat tolerance, and improved fracture toughness. To shed light on the electronic mechanism that enables transformation-induced plasticity (Fig. 1 and fig. S2)—here argued to be the atomic-scale origin of enhanced fracture resistance (Figs. 2 and 3)—we analyze densities of states (DOS) and energy-resolved electron densities of HEC supercell models subject to tensile strain. We focus on [001] strain because, irrespective of the elongation direction, {001} surfaces are the most likely fracture planes (see table S1 and pink-framed insets in Fig. 1 and fig. S2). For our comparative analysis, we consider (Ti,Zr,Hf,Ta,W)C and (V,Ta,Cr,Mo,W)C as representative cases of low and high-VEC compositions.

Figure 5 shows total and site *lm*–resolved electronic DOS of unstrained and strained (V,Ta,Cr,Mo,W)C and (Ti,Zr,Hf,Ta,W)C lattices at 300 K. The Fermi energies (E_F) are indicated by black dashed vertical lines. Since high-VEC alloys are prone to transform plastically, the DOS of (V,Ta,Cr,Mo,W)C is computed every 30 AIMD ionic steps to ensure that the DOS profile remains constant at a given strain. Thus, the plots in Fig. 5 (A to C) are the result of superposed DOS curves, as evidenced by the line-broadening. Conversely, the low-VEC (Ti,Zr,Hf,Ta,W)C maintains sixfold octahedral coordination up to fracture. For this material, it is sufficient to calculate the DOS at a single AIMD time step at 0 strain (Fig. 5D) and at 10% strain (Fig. 5E). In Fig. 5E, the DOS is computed at an AIMD time step before initiation of fracture. The orange dotted vertical lines in Fig. 5 (A to C) mark the position of a pseudo-gap, which serves for further electronic structure analysis (see below). In B1 structure carbonitrides, the pseudo-gap energy

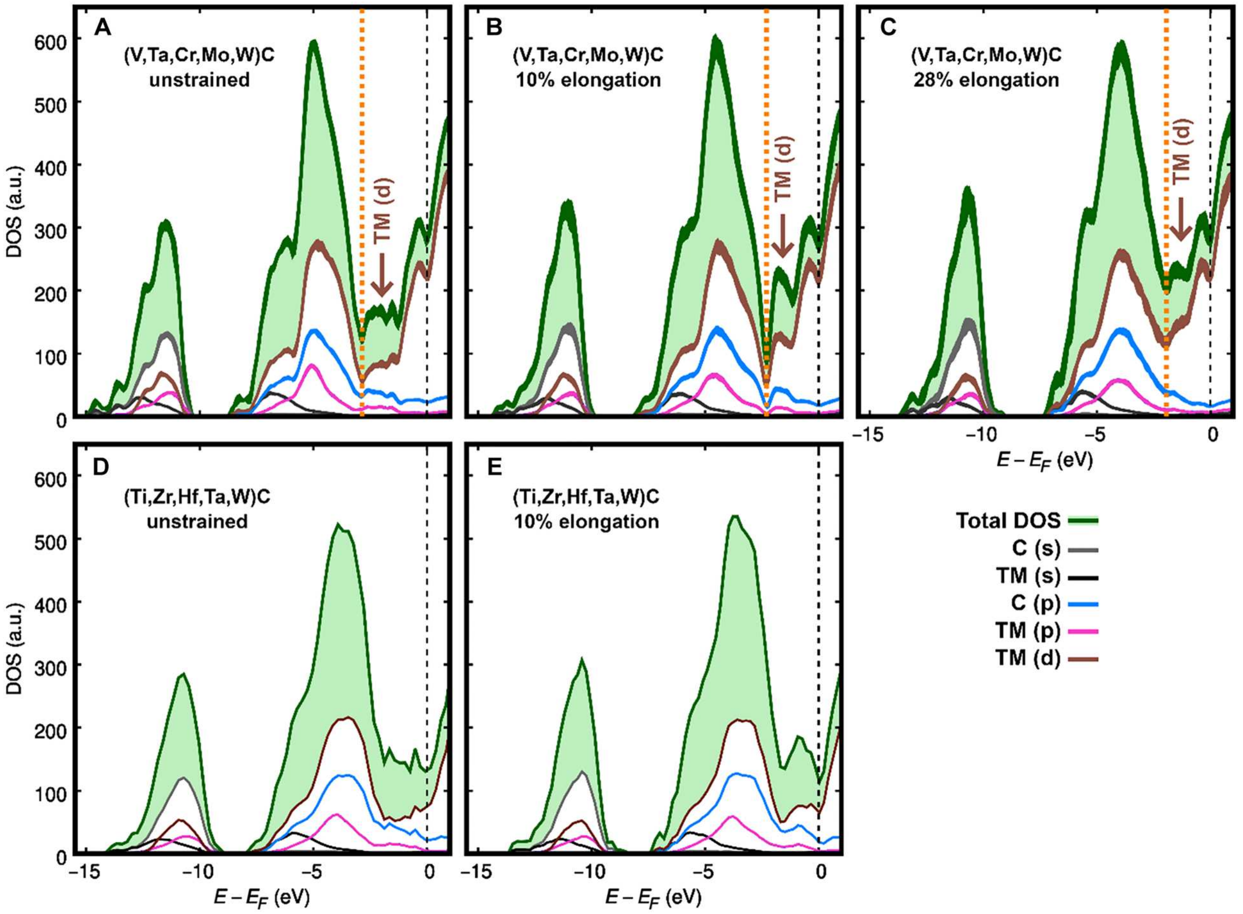

Fig. 5. DOS comparison for low- and high-VEC HECs. Spin-up + spin-down electronic DOS of (V,Ta,Cr,Mo,W)C (**A** to **C**) and (Ti,Zr,Hf,Ta,W)C (**D** and **E**) calculated at different tensile strains. The Fermi energy E_F is at 0 eV. For (V,Ta,Cr,Mo,W)C, the DOS is calculated every 30 fs: line broadening due to small fluctuations of DOS curves over time. For (Ti,Zr,Hf,Ta,W)C—which fractures at 10% strain—the DOS in (E) is computed at a time step before bond breakage. The site-projected DOS (TM, transition metals; C, carbon) resolved in *l,m* quantum numbers are colored as indicated in the legend. The labels with brown arrows "TM (d) →" near Fermi levels in (A) to (C) indicate the substantial change in the hybridization of d electrons during deformation and plastic transformation of (V,Ta,Cr,Mo,W)C (note the rise of the brown-colored d DOS curves). a.u., arbitrary units.

separates electronic states primarily used in nearest-neighbor (metal-C) or next-nearest-neighbor (metal-metal) bonds (*13*, *15*, *16*).

The DOS peaks at energy intervals from −15 to −10 eV for (V,Ta,Cr,Mo,W)C (Fig. 5A) and from −14 to −9 eV for (Ti,Zr,Hf,Ta,W)C (Fig. 5D) are primarily due to carbon (s) states. The p-d states of strong carbon-TM bonds, responsible for the intrinsic hardness of B1 TM carbides, are mostly contained in energy ranges between −8 eV and a pseudo-gap. The pseudo-gap energy coincides with a local dip in the density of d states (brown color curves in Fig. 5). For unstrained (V,Ta,Cr,Mo,W)C, the pseudo-gap position is marked by the orange dotted vertical line at −3 eV in Fig. 5A. For unstrained (Ti,Zr,Hf,Ta,W)C (Fig. 5D), the pseudo-gap is ≈2 eV below the Fermi energy. The electronic states enclosed between the pseudo-gap and the Fermi level have mainly d character (*13*, *16*). These states are particularly sensitive to the deformation of the material. For example, the enhanced population of TM (d) − TM (d) states results in reduced shear resistance, which is predicted to improve ductility in B1 TM carbonitrides (*12*, *13*).

The pseudo-gap shifts to energies closer to the Fermi level as the strain increases. For (V,Ta,Cr,Mo,W)C, the pseudo-gap energy increases from −2.9 eV (unstrained Fig. 5A) to −2.3 eV (10% elongation Fig. 5B) and to −2.0 eV at 28% elongation (Fig. 5C). The latter is the maximum strain withstood by (V,Ta,Cr,Mo,W)C before rupture (Fig. 1). The change in pseudo-gap position is accompanied by a notable increase in density of d electronic states (see brown arrows in Fig. 5, A to C). Although less pronounced, a similar effect can be seen for (Ti,Zr,Hf,Ta,W)C by comparing the d electron DOS near the Fermi level of Fig. 5 (D and E). Substantial strain-induced modifications of the DOS of (V,Ta,Cr,Mo,W)C in comparison to (Ti,Zr,Hf,Ta,W)C suggest that rehybridization of d states occurs more easily in high-VEC alloys. The phenomenon assists in the reorganization of the bonding network to comply with deformation. This possibility appears to be precluded for (Ti,Zr,Hf,Ta,W)C, as the change in d electron DOS peaks is more modest (Fig. 5, D and E) and the material cleaves at 10% elongation (Fig. 1). Accordingly, we infer that low-VEC HECs are more susceptible to brittle fracture due to low ability to delocalize d electrons during deformation.

The energy-resolved electron density of high-VEC (V,Ta,Cr,-Mo,W)C is plotted in Fig. 6. The energy ranges used for electron density calculations are between the pseudo-gap (orange dotted lines in Fig. 5, A to C) and the Fermi energy, specifically [−2.9 eV, E_F] for the unstrained structure, [−2.3 eV, E_F] for 10%, and [−2.0 eV, E_F] for 28% strain, respectively. Within these energy intervals, the electron densities are primarily due to d states. The proportion between densities of electrons with s, p, and d characters can be assessed by comparing the areas of corresponding DOS curves near the Fermi level in Fig. 5 (A to C). To facilitate interpretation, Fig. 6 shows a small region of the entire charge density map. Complete electron density distributions on (100) and (110) planes parallel to the elongation direction can be found in fig. S7.

In Fig. 6, low and high electron densities are colored red-yellow and blue-purple, respectively. A high concentration of blue-purple color on atomic sites indicates that the majority of valence electrons reside near the nuclei. As the strain increases (Fig. 6, B and C), yellow-red electron-depleted areas appear along lattice planes orthogonal to the strain direction. The observed electron depletion is caused by the breakage of some bonds parallel to elongation. At the same time, however, part of the electrons transfers into d-d bonds between next-nearest-neighbor metal atoms. The effect is indicated by black dotted lines that point at electron accumulation (blue color) between TM atoms in Fig. 6 (B and C). The phenomenon prevents fracture by assisting modifications in bond angles and atomic coordination. To summarize, the ability of high-VEC HECs to redistribute electrons during deformation enables transformation plasticity, which enhances the material resistance to fracture at a low expense in hardness. The effectiveness of electronic responses in assisting transformation toughening may depend on the strain direction. Nevertheless, Figs. 5 and 6 show that electron rehybridization operates during strain orthogonal to {001} surfaces: easiest fracture planes in B1 structure HECs.

Deviations of mechanical properties from theoretical trends and future research directions

This work goes beyond ab initio–predicted trends in mechanical properties versus VEC for ceramics to offer unprecedented insights on electronic effects and associated transformation plasticity mechanisms correlating with an intrinsically (statistically) higher resistance to fracture of HEC ceramics. However, we should briefly discuss other effects that—along with the VEC—influence hardness and intragrain resistance to fracture of TM carbide and nitride ceramics. Below, we provide a few examples of the limitations inherent to this study.

Lattice vacancies may positively or negatively affect the hardness of TM carbides (*49*, *53*). Modulations in vacancy contents across the sample may locally promote the formation of secondary phases or vacancy-ordered phases with mechanical properties different from disordered B1 solid solutions (*54*–*56*). Besides point defects, the density of stacking faults influences mechanical properties. The density of stacking faults in B1 structure TM carbonitrides can also be tailored via VEC tuning. For example, it has been shown that group V carbides (VEC = 9) have an intrinsically higher density of stacking faults than group IV carbides (VEC = 8) (*57*). Notably, Ta substitutions in (Hf,Ta)C alloys may promote slip on {111} planes because they produce metastable stacking fault configurations (*18*). The observation is consistent with pioneering studies on group V carbides that inferred preferential slip on {111} planes via analysis of hardness anisotropy (*58*, *59*). Recent micropillar compression experiments also show increased dislocation mobility on {111} planes of group V, in comparison to group IV, carbides (*32*, *33*, *60*). We note that lattice slip on {111} planes may improve ductility by providing enough independent slip systems, according to criteria by von Mises (*61*). However, stacking faults can also contribute to hardening the material. Hugosson *et al.* (*62*) demonstrated that TM carbide and nitride alloys with VEC near 9.5 have an

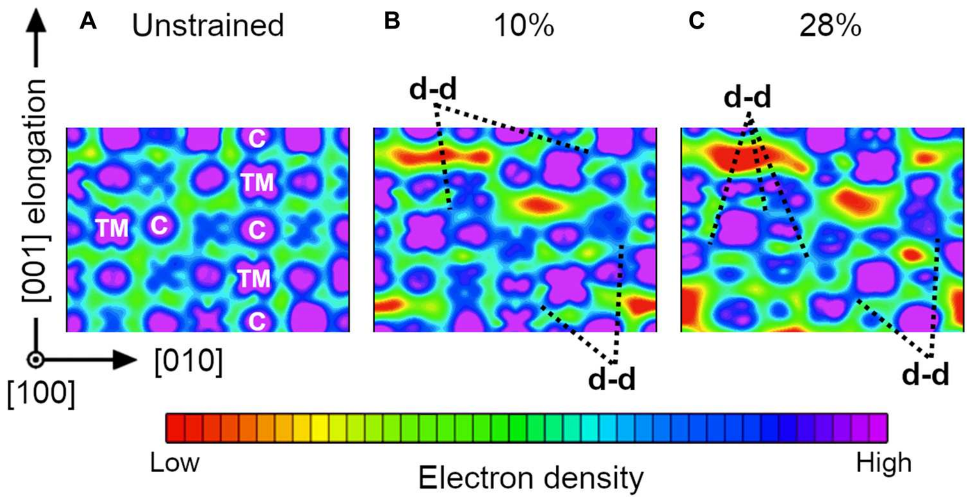

Fig. 6. Changes in charge density during deformation. Energy-resolved electron density of B1 (V,Ta,Cr,Mo,W)C calculated at supercell [001] elongations of 0 (**A**), 10 (**B**), and 28% (**C**). The charge density cut is on a (100) lattice plane parallel to the elongation direction. On {001} planes, cation and anion sites have chessboard arrangement: note carbon and metal atoms labeled with "C" and "TM" in (A). The energy ranges used for electron density calculations are between the pseudo-gap (indicated by orange dotted lines in Fig. 5, A to C) and the Fermi energy. For a fair comparison, each electron density ρ is normalized by the total number of electrons included in the corresponding energy range. A logarithmic color scale is used to facilitate visualization of strain-induced changes in electron arrangements. Off-scale electron densities are colored in red and purple. Note that this figure depicts a portion of the entire supercell. The full figure (fig. S7), with detailed information for the color legend, is presented in the Supplementary Materials.

equal energetic preference for cubic and hexagonal phases (*63*). This favors nucleation of {111} stacking faults during the growth of B1 structure alloys and enhances hardness by hindering dislocation motion across the fault (*62*, *63*). Although here neglected, point defects and extended crystallographic defects surely affect the mechanical properties of HECs and cause deviations from theoretical trends that are exclusively based on properties of defect-free crystals.

The results of this work may inspire experimental studies (e.g., in-depth transmission electron microscopy) and theoretical developments dedicated to locating stress-induced nanoscale transformations and quantifying the influence of such transformations on the fracture toughness of HECs. Material selection for individual applications will necessitate further investigation into the long-term durability and stability of these HECs when considering the operating environment (e.g., thermal cycling, chemical environment, or oxidation resistance). For example, prior work in a selection of HECs for oxidative environments suggests considerable ability to modulate the oxidation rate based on individual element selection (*4*). It is possible that the suitability for other applications could be tuned similarly, perhaps while maintaining a preferred VEC.

High-entropy ceramics offer a wide compositional playground for tuning VEC and structural stability. For example, the combination of nitrogen and carbon may facilitate the stabilization of single-phase B1 structure ceramics containing TMs other than the refractory ones, while also contributing to enriching the phase diagram with competing polymorph structures (see, e.g., graphitic-like domains nucleating in high-VEC HECs subject to [110] strain in the inset in fig. S2). In parallel to the experimental exploration of unprecedented HEC compositions, it would be desirable to perform larger scale (domain sizes between nm^3 and μm^3) atomistic simulations over longer time frames (≤microseconds). These would allow us to shed light on effects induced on mechanical properties by native extended crystallographic defects (as well as their formation and interactions) during the deformation of pristine or flawed ceramics. Reliable atomistic description of nano- and microscale properties in HECs will soon become possible thanks to the rapid development of machine learning interatomic potentials (MLIPs), which combine accuracy comparable to the underlying ab initio training set to the efficiency of semi-empirical model interactions. MLIP-based computations and simulations will enable quantitatively predictive mechanical testing and reliable evaluation of K_{Ic} [as prescribed by rigorous theoretical approaches, see, e.g., (*64*, *65*)] while providing direct visualization of nanoscale phenomena that are inaccessible for computationally intensive ab initio methods.

In summary, we have investigated the hardness and intragrain fracture resistance of B1 structure refractory HECs as a function of the alloy VEC. AIMD simulations at room temperature show that the alloy tensile toughness U_T increases substantially for HECs with VEC $\gtrsim$ 9.4 e^-/f.u. (here named high-VEC HECs) due to strain-activated transformation plasticity that prevents brittle fracture. The theoretical trends correlate with the statistics collected by nanoindentation experiments accounting for both loading force and penetration depth. Our results demonstrate enhanced resistance to fracture of severely loaded high-VEC alloys in comparison to HECs with VEC ≤ 9 e^-/f.u. (low-VEC HECs). In high-VEC alloys, the superior fracture resistance comes at a modest expense in hardness and theoretical strength, which remain comparable to those of low-VEC ceramics and other TM carbides. Via analyses of electronic structures, we propose that a high VEC in HECs enables electron reorganization during deformation, which dissipates accumulated stress by allowing for local changes in bond angles and atomic coordination. The insight gleaned from this work offers a rationale for the design of refractory HEC alloys that combine high hardness and heat tolerance to improved toughness. We infer that HECs with VEC $\gtrsim$ 9.4 e^-/f.u. are generally expected to exhibit such an excellent combination of properties.

MATERIALS AND METHODS

First principles

Born Oppenheimer AIMD simulations of HEC single-crystal models subject to tensile (and shear) deformation are carried out at room temperature within the NVT ensemble using the Nosé-Hoover thermostat. The simulations are based on Kohn-Sham DFT with the generalized gradient approximation for electronic exchange and correlation energies parameterized by Perdew, Burke, and Ernzerhof (*66*) and the projector augmented wave method, as implemented in VASP (Vienna Ab initio Simulation Package) (*67*–*69*). The equations of motion are integrated on 1-fs time steps, with forces calculated by Γ-point sampling and 300-eV cutoff energies for the basis set. Supercell elongation is applied orthogonal to (001) and (110) surfaces. The surface energies E_{surf} of two representative HEC compositions (one low-VEC and one high-VEC) are calculated by DFT, as described in the Supplementary Materials. Details of AIMD mechanical testing simulations can be found in (*22*, *26*). Briefly, the shape and orientation of [001] and [110] vertically oriented supercells used for tensile testing simulations (576 atoms in all cases) can be seen in figure 1 (A and B) of (*22*). Before deformation, the equilibrium supercell structures and lattice parameters are determined via NPT sampling using the Parrinello-Rahman barostat and Langevin thermostat. Strain is sequentially increased by 2%, equilibrating the structure at each step during ≈3 ps. The strain is incremented until the occurrence of fracture. Different from our previous studies, here, we also consider Cr substitutions, which produce magnetic moments in the alloy. We treat the electronic-spin degrees of freedom via the disordered local moment approximation, which has been previously used to model the paramagnetic state of, e.g., B1 structure CrN by AIMD simulations at finite temperatures (*70*). Collinear magnetic moments are randomly reinitialized to ±2 μ_B every 30 time steps. At every strain step, Cr-containing supercells are thermally equilibrated for ≈1.0 ps. Time- and space-averaged total magnetizations are close to zero. AIMD movies of B1 structure HECs are generated using VMD (Visual Molecular Dynamics) (*71*).

Sample preparation

Six five-cation HECs were prepared with VEC ranging from 8.4 to 9.6. The nominal composition of each material was prepared starting from the individual binary carbides (Cr_3C_2, HfC, Mo_2C, NbC, TaC, TiC, VC, W_2C, and ZrC,) in >99% purity and −325 mesh (Alfa Aesar). High energy ball milling of each sample is performed in tungsten carbide–lined steel jars with tungsten carbide milling media and in an inert argon environment for a total of 2 hours. Overheating and oxidation are further avoided by ball milling in 30-min intervals with 15-min breaks. Bulk samples are simultaneously densified and homogenized using the solid-state processing

technique commonly referred to as spark plasma sintering (Thermal Technologies, CA, USA), also known as the field-assisted–sintering technique. Sintering is performed in graphite dies at 2473 K with a heating rate of 100 K/min, 30-MPa uniaxial pressure applied at temperature, and a 10-min dwell. For all samples, the sintering process is performed under a vacuum. The final sample dimensions are 20 mm in diameter and approximately 5 mm thick.

X-ray diffraction and electron microscopy

Crystal structure analysis is performed using a Rigaku Miniflex equipped with a one-dimensional detector. Diffraction data are collected from 2θ in the range of 20° to 120° using a 0.02° step size and a scan rate of 5°/min. Copper Kα radiation is used for all x-ray diffraction measurements. Sample densities from the Archimedes principle are compared to the theoretical density determined by the respective x-ray diffraction patterns before nanoindentation to minimize the effect of porosity. An Apreo (Thermo Fisher Scientific) scanning electron microscope operating at 20 kV is used to evaluate the location (e.g., not on grain boundaries or pores) and cracked state of individual nanoindentations. The EBSD camera is an Oxford Symmetry complementary metal-oxide semiconductor (CMOS) detector and the data were collected using a 1-μm step size.

Nanoindentation

Nanoindentation is carried out using a KLA-Tencor G200 Nanoindenter (KLA-Tencor, CA, USA). Hardness measurements are performed in accordance with ISO standard 1477 in the range of 50 to 500 mN in 50 mN increments. The nanoindentation indents are spaced 50 μm apart to avoid stress effects from nearby indents. The nanoindentation arrays are performed to collect statistics on the number of nanoindents that result in observable cracks, with respect to load and indentation depth, as a function of the material's VEC. Penetration depth data are aggregated by binning the recorded indentation depths during force-controlled nanoindentation. Statistically significant results regarding the bulk material's mechanical behavior are ensured by collecting many valid (i.e., not at a grain boundary nor located against a pore) data points from diverse crystallographic orientations. Refer to (*26*) for examples of indents exhibiting cracks or not. Here, at least 28 indents per load per sample remained after filtering out invalid data points. The average number of valid indents per load per sample was 45 with a standard deviation of 11. This resulted in a minimum of 337 and a maximum of 600 (average of 449 ± 107) total usable data points per sample.

Supplementary Materials

This PDF file includes:
Figs. S1 to S7
Tables S1 to S3
Legends for movies S1 to S4

Other Supplementary Material for this manuscript includes the following:
Movies S1 to S4

REFERENCES AND NOTES

1. C. Oses, C. Toher, S. Curtarolo, High-entropy ceramics. *Nat. Rev. Mater.* **5**, 295–309 (2020).
2. O. F. Dippo, D. G. Sangiovanni, E. Wenger, K. S. Vecchio, Color and pseudogap tunability in multicomponent carbonitrides. *Mater. Des.* **217**, 110600 (2022).
3. J. Sun, L. Guo, Y. Zhang, Y. Wang, K. Fan, Y. Tang, Superior phase stability of high entropy oxide ceramic in a wide temperature range. *J. Eur. Ceram. Soc.* **42**, 5053–5064 (2022).
4. W. M. Mellor, K. Kaufmann, O. F. Dippo, S. D. Figueroa, G. D. Schrader, K. S. Vecchio, Development of ultrahigh-entropy ceramics with tailored oxidation behavior. *J. Eur. Ceram. Soc.* **41**, 5791–5800 (2021).
5. K. Kaufmann, D. Maryanovsky, W. M. Mellor, C. Zhu, A. S. Rosengarten, T. J. Harrington, C. Oses, C. Toher, S. Curtarolo, K. S. Vecchio, Discovery of high-entropy ceramics via machine learning. *Npj Comput. Mater.* **6**, 42 (2020).
6. P. Sarker, T. Harrington, C. Toher, C. Oses, M. Samiee, J. P. Maria, D. W. Brenner, K. S. Vecchio, S. Curtarolo, High-entropy high-hardness metal carbides discovered by entropy descriptors. *Nat. Commun.* **9**, 4980 (2018).
7. J. Gild, Y. Zhang, T. Harrington, S. Jiang, T. Hu, M. C. Quinn, W. M. Mellor, N. Zhou, K. Vecchio, J. Luo, High-entropy metal diborides: A new class of high-entropy materials and a new type of ultrahigh temperature ceramics. *Sci. Rep.* **6**, 37946 (2016).
8. J. Gild, J. Braun, K. Kaufmann, E. Marin, T. Harrington, P. Hopkins, K. Vecchio, J. Luo, A high-entropy silicide: $(Mo_{0.2}Nb_{0.2}Ta_{0.2}Ti_{0.2}W_{0.2})Si_2$. *J. Mater.* **5**, 337–343 (2019).
9. T. J. Harrington, J. Gild, P. Sarker, C. Toher, C. M. Rost, O. F. Dippo, C. McElfresh, K. Kaufmann, E. Marin, L. Borowski, P. E. Hopkins, J. Luo, S. Curtarolo, D. W. Brenner, K. S. Vecchio, Phase stability and mechanical properties of novel high entropy transition metal carbides. *Acta Mater.* **166**, 271–280 (2019).
10. D. G. Pettifor, Theoretical predictions of structure and related properties of intermetallics. *Mater. Sci. Technol.* **8**, 345–349 (1992).
11. S. F. Pugh, XCII. Relations between the elastic moduli and the plastic properties of polycrystalline pure metals. *Phil. Mag.* **45**, 823–843 (1954).
12. K. Balasubramanian, S. V. Khare, D. Gall, Valence electron concentration as an indicator for mechanical properties in rocksalt structure nitrides, carbides and carbonitrides. *Acta Mater.* **152**, 175–185 (2018).
13. D. G. Sangiovanni, L. Hultman, V. Chirita, Supertoughening in B1 transition metal nitride alloys by increased valence electron concentration. *Acta Mater.* **59**, 2121–2134 (2011).
14. D. Edström, D. G. Sangiovanni, L. Hultman, I. Petrov, J. E. Greene, V. Chirita, Elastic properties and plastic deformation of TiC- and VC-based pseudobinary alloys. *Acta Mater.* **144**, 376–385 (2018).
15. S. H. Jhi, J. Ihm, S. G. Louie, M. L. Cohen, Electronic mechanism of hardness enhancement in transition-metal carbonitrides. *Nature* **399**, 132–134 (1999).
16. H. Kindlund, D. G. Sangiovanni, I. Petrov, J. E. Greene, L. Hultman, A review of the intrinsic ductility and toughness of hard transition-metal nitride alloy thin films. *Thin Solid Films* **688**, 137479 (2019).
17. D. G. Sangiovanni, L. Hultman, V. Chirita, I. Petrov, J. E. Greene, Effects of phase stability, lattice ordering, and electron density on plastic deformation in cubic TiWN pseudobinary transition-metal nitride alloys. *Acta Mater.* **103**, 823–835 (2016).
18. C. J. Smith, X.-X. Yu, Q. Guo, C. R. Weinberger, G. B. Thompson, Phase, hardness, and deformation slip behavior in mixed $Hf_xTa_{1-x}C$. *Acta Mater.* **145**, 142–153 (2018).
19. H. Kindlund, D. G. Sangiovanni, L. Martinez-de-Olcoz, J. Lu, J. Jensen, J. Birch, I. Petrov, J. E. Greene, V. Chirita, L. Hultman, Toughness enhancement in hard ceramic thin films by alloy design. *APL Mater.* **1**, 042104 (2013).
20. M. Schloegl, C. Kirchlechner, J. Paulitsch, J. Keckes, P. H. Mayrhofer, Effects of structure and interfaces on fracture toughness of CrN/AlN multilayer coatings. *Scr. Mater.* **68**, 917–920 (2013).
21. K. Yalamanchili, I. C. Schramm, E. Jimenez-Pique, L. Rogstrom, F. Mucklich, M. Oden, N. Ghafoor, Tuning hardness and fracture resistance of $ZrN/Zr_{0.63}Al_{0.37}N$ nanoscale multilayers by stress-induced transformation toughening. *Acta Mater.* **89**, 22–31 (2015).
22. D. G. Sangiovanni, F. Tasnadi, L. J. S. Johnson, M. Oden, I. A. Abrikosov, Strength, transformation toughening, and fracture dynamics of rocksalt-structure $Ti_{1-x}Al_xN$ ($0 \le x \le 0.75$) alloys. *Phys. Rev. Mater.* **4**, 033605 (2020).
23. M. Bartosik, C. Rumeau, R. Hahn, Z. L. Zhang, P. H. Mayrhofer, Fracture toughness and structural evolution in the TiAlN system upon annealing. *Sci. Rep.* **7**, 16476 (2017).
24. N. Koutná, L. Löfler, D. Holec, Z. Chen, Z. Zhang, L. Hultman, P. H. Mayrhofer, D. G. Sangiovanni, Atomistic mechanisms underlying plasticity and crack growth in ceramics: A case study of AlN/TiN superlattices. *Acta Mater.* **229**, 117809 (2022).
25. H. Levamaki, F. Tasnadi, D. G. Sangiovanni, L. J. S. Johnson, R. Armiento, I. A. Abrikosov, Predicting elastic properties of hard-coating alloys using ab-initio and machine learning methods. *Npj Comput. Mater.* **8**, 17 (2022).
26. D. G. Sangiovanni, W. Mellor, T. Harrington, K. Kaufmann, K. Vecchio, Enhancing plasticity in high-entropy refractory ceramics via tailoring valence electron concentration. *Mater. Des.* **209**, 109932 (2021).
27. W. Liu, X. Liu, W. T. Zheng, Q. Jiang, Surface energies of several ceramics with NaCl structure. *Surf. Sci.* **600**, 257–264 (2006).

28. D. G. Sangiovanni, F. Tasnádi, T. Harrington, M. Odén, K. S. Vecchio, I. A. Abrikosov, Temperature-dependent elastic properties of binary and multicomponent high-entropy refractory carbides. *Mater. Des.* **204**, 109634 (2021).
29. P. Steneteg, O. Hellman, O. Y. Vekilova, N. Shulumba, F. Tasnadi, I. A. Abrikosov, Temperature dependence of TiN elastic constants from ab initio molecular dynamics simulations. *Phys. Rev. B.* **87**, 094114 (2013).
30. O. F. Dippo, N. Mesgarzadeh, T. J. Harrington, G. D. Schrader, K. S. Vecchio, Bulk high-entropy nitrides and carbonitrides. *Sci. Rep.* **10**, 21288 (2020).
31. K. Kaufmann, E. Wenger, K. Vecchio, Synthesis and evaluation of rocksalt structure (Mo, W) carbides via vanadium additions. *Scr. Mater.* **229**, 115382 (2023).
32. A. Aleman, H. Zaid, B. M. Cruz, K. Tanaka, J. M. Yang, H. Kindlund, S. Kodambaka, Room-temperature plasticity and size-dependent mechanical responses in small-scale B1-NbC (001) single-crystals. *Acta Mater.* **221**, 117384 (2021).
33. S. Kiani, J.-M. Yang, S. Kodambaka, Nanomechanics of refractory transition-metal carbides: A path to discovering plasticity in hard ceramics. *J. Am. Ceram. Soc.* **98**, 2313–2323 (2015).
34. H. Waldl, M. Tkadletz, A. Lechner, C. Czettl, M. Pohler, N. Schalk, Evolution of the fracture properties of arc evaporated $Ti_{1-x}Al_xN$ coatings with increasing Al content. *Surf. Coat. Technol.* **444**, 128690 (2022).
35. A. B. Mei, H. Kindlund, E. Broitman, L. Hultman, I. Petrov, J. E. Greene, D. G. Sangiovanni, Adaptive hard and tough mechanical response in single-crystal B1 VN_x ceramics via control of anion vacancies. *Acta Mater.* **192**, 78–88 (2020).
36. J. Salamania, D. G. Sangiovanni, A. Kraych, K. M. C. Kwick, I. C. Schramm, L. J. S. Johnson, R. Boyd, B. Bakhit, T. W. Hsu, M. Mrovec, L. Rogström, F. Tasnadi, I. A. Abrikosov, M. Oden, Elucidating dislocation core structures in titanium nitride through high-resolution imaging and atomistic simulations. *Mater. Des.* **224**, 111327 (2022).
37. S. K. Yadav, R. Ramprasad, A. Misra, X. Y. Liu, Core structure and Peierls stress of edge and screw dislocations in TiN: A density functional theory study. *Acta Mater.* **74**, 268–277 (2014).
38. W. J. Clegg, Controlling cracks in ceramics. *Science* **286**, 1097–1099 (1999).
39. P. M. Kelly, L. R. F. Rose, The martensitic transformation in ceramics–Its role in transformation toughening. *Prog. Mater. Sci.* **47**, 463–557 (2002).
40. R. H. J. Hannink, P. M. Kelly, B. C. Muddle, Transformation toughening in zirconia-containing ceramics. *J. Am. Ceram. Soc.* **83**, 461–487 (2000).
41. P. L. Larsson, A. E. Giannakopoulos, Tensile stresses and their implication to cracking at pyramid indentation of pressure-sensitive hard metals and ceramics. *Mater. Sci. Eng. A. Struct.* **254**, 268–281 (1998).
42. M. Trunec, Z. Chlup, Higher fracture toughness of tetragonal zirconia ceramics through nanocrystalline structure. *Scr. Mater.* **61**, 56–59 (2009).
43. A. Bravo-Leon, Y. Morikawa, M. Kawahara, M. J. Mayo, Fracture toughness of nanocrystalline tetragonal zirconia with low yttria content. *Acta Mater.* **50**, 4555–4562 (2002).
44. G. N. Morscher, P. Pirouz, A. H. Heuer, Temperature-dependence of hardness in yttria-stabilized zirconia single-crystals. *J. Am. Ceram. Soc.* **74**, 491–500 (1991).
45. R. G. Wellman, A. Dyer, J. R. Nicholls, Nano and micro indentation studies of bulk zirconia and EB PVD TBCs. *Surf. Coat. Technol.* **176**, 253–260 (2004).
46. M. Sygnatowicz, R. A. Cutler, D. K. Shetty, ζ-Ta_4C_{3-x}: A high fracture toughness carbide with rising-crack-growth-resistance (R-curve) behavior. *J. Am. Ceram. Soc.* **98**, 2601–2608 (2015).
47. G. D. Quinn, Fracture Toughness of Ceramics by the Vickers Indentation Crack Length Method: A Critical Review. *Ceramic Engineering and Science Proceedings* 27, 45–62 (2007).
48. S. Zhang, D. Sun, Y. Q. Fu, H. J. Du, Toughness measurement of thin films: A critical review. *Surf. Coat. Technol.* **198**, 74–84 (2005).
49. H. Holleck, Material selection for hard coatings. *J. Vac. Sci. Technol. A* **4**, 2661–2669 (1986).
50. E. J. Pavlina, C. J. Van Tyne, Correlation of yield strength and tensile strength with hardness for steels. *J. Mater. Eng. Perform.* **17**, 888–893 (2008).
51. M. A. Salazar-Guapuriche, Y. Y. Zhao, A. Pitman, A. Greene, Correlation of strength with hardness and electrical conductivity for aluminium alloy 7010. *Mater. Sci. Forum* **519-521**, 853–858 (2006).
52. S. H. Hashemi, Strength-hardness statistical correlation in API X65 steel. *Mater. Aci. Eng. A. Struct.* **528**, 1648–1655 (2011).
53. X.-X. Yu, G. B. Thompson, C. R. Weinberger, Influence of carbon vacancy formation on the elastic constants and hardening mechanisms in transition metal carbides. *J. Eur. Ceram. Soc.* **35**, 95–103 (2015).
54. H. Yu, G. B. Thompson, C. R. Weinberger, The role of chemistry and bonding in regulating fracture in multiphase transition metal carbides and nitrides. *Extreme Mech. Lett.* **17**, 1–6 (2017).
55. C. R. Weinberger, G. B. Thompson, Review of phase stability in the group IVB and VB transition-metal carbides. *J. Am. Ceram. Soc.* **101**, 4401–4424 (2018).
56. H. Yu, M. Guziewski, G. B. Thompson, C. R. Weinberger, A model for understanding the formation energies of nanolamellar phases in transition metal carbides and nitrides. *Model. Simul. Mater. Sci. Eng.* **24**, 055004 (2016).
57. N. De Leon, X. X. Yu, H. Yu, C. R. Weinberger, G. B. Thompson, Bonding effects on the slip differences in the B1 monocarbides. *Phys. Rev. Lett.* **114**, 165502 (2015).
58. R. H. J. Hannink, D. L. Kohlstedt, M. J. Murray, Slip system determination in cubic carbides by hardness anisotropy. *Proc. R. Soc. Lond. A* **326**, 409–420 (1972).
59. D. J. Rowcliffe, G. E. Hollox, Hardness anisotropy, deformation mechanisms and brittle-to-ductile transition in carbide. *J. Mater. Sci.* **6**, 1270–1276 (1971).
60. S. Kiani, C. Ratsch, A. M. Minor, J. M. Yang, S. Kodambaka, In situ transmission electron microscopy observations of room-temperature plasticity in sub-micron-size TaC(100) and TaC(011) single crystals. *Scr. Mater.* **100**, 13–16 (2015).
61. R. von Mises, Mechanik der plastischen Formänderung von Kristallen. *Z. Angew. Math. Mech.* **8**, 161–185 (1928).
62. H. W. Hugosson, U. Jansson, B. Johansson, O. Eriksson, Restricting dislocation movement in transition metal carbides by phase stability tuning. *Science* **293**, 2434–2437 (2001).
63. T. Joelsson, L. Hultman, H. W. Hugosson, J. M. Molina-Aldareguia, Phase stability tuning in the $Nb_xZr_{1-x}N$ thin-film system for large stacking fault density and enhanced mechanical strength. *Appl. Phys. Lett.* **86**, 131922 (2005).
64. P. Andric, W. A. Curtin, New theory for mode I crack-tip dislocation emission. *J. Mech. Phys. Solids* **106**, 315–337 (2017).
65. P. Andric, W. A. Curtin, Atomistic modeling of fracture. *Model. Simul. Mater. Sci. Eng.* **27**, 013001 (2019).
66. J. P. Perdew, K. Burke, M. Ernzerhof, Generalized gradient approximation made simple. *Phys. Rev. Lett.* **77**, 3865–3868 (1996).
67. G. Kresse, D. Joubert, From ultrasoft pseudopotentials to the projector augmented-wave method. *Phys. Rev. B* **59**, 1758–1775 (1999).
68. G. Kresse, J. Hafner, Ab initio molecular dynamics for liquid metals. *Phys. Rev. B* **47**, 558–561 (1993).
69. G. Kresse, J. Furthmuller, Efficiency of ab-initio total energy calculations for metals and semiconductors using a plane-wave basis set. *Comput. Mater. Sci.* **6**, 15–50 (1996).
70. N. Shulumba, B. Alling, O. Hellman, E. Mozafari, P. Steneteg, M. Oden, I. A. Abrikosov, Vibrational free energy and phase stability of paramagnetic and antiferromagnetic CrN from ab initio molecular dynamics. *Phys. Rev. B* **89**, 174108 (2014).
71. W. Humphrey, A. Dalke, K. Schulten, VMD: Visual molecular dynamics. *J. Mol. Graph. Model.* **14**, 33–38 (1996).

Acknowledgments: We would like to thank M. Qin for assistance with nanoindentation and analysis of the samples. All simulations were carried out using the resources provided by the Swedish National Infrastructure for Computing (SNIC)—partially funded by the Swedish Research Council through grant agreement no. VR-2018-05973. **Funding:** This work was supported by Swedish Research Council (VR) grant VR-2021-04426 (to D.G.S.); Competence Center Functional Nanoscale Materials (FunMat-II) grant 2022-03071 (to D.G.S.); Olle Engkvist Foundation (to D.G.S.); Department of NanoEngineering's Materials Research Center (NEMRC), UC San Diego (to K.V. and K.K.); National Defense Science and Engineering Graduate (NDSEG) Fellowship Program (to K.K.); ARCS Foundation, San Diego Chapter (to K.K.), and The Oerlikon Group (to K.V.). **Author contributions:** Conceptualization: D.G.S., K.V., and K.K. Methodology: D.G.S., K.K., and K.V. Modeling: D.G.S. Experimental: K.K. Visualization: D.G.S. and K.K. Writing—original draft: D.G.S. Writing—review and editing: K.K. and K.V. **Competing interests:** The authors declare that they have no competing interests. **Data and materials availability:** All data needed to evaluate the conclusions in the paper are present in the paper and/or the Supplementary Materials.

Submitted 17 April 2023
Accepted 10 August 2023
Published 13 September 2023
10.1126/sciadv.adi2960
