# Supplementary material for: Valence electron concentration as key parameter to control the fracture resistance of refractory high-entropy carbides
Source: Sci Adv. 2023 Sep 13;9(37):eadi2960. doi: 10.1126/sciadv.adi2960 (PMC10499311; doi:10.1126/sciadv.adi2960)
Supplement: Supplementary file 2 — Figs. S1 to S7 Tables S1 to S3 Legends for movies S1 to S4 [file sciadv.adi2960_sm.v2.pdf]

CORRECTED 19 JANUARY 2024

Science Advances
AAAS

# Supplementary Materials for

## Valence electron concentration as key parameter to control the fracture resistance of refractory high-entropy carbides

Davide G. Sangiovanni *et al.*

Corresponding author: Kenneth Vecchio, kvecchio@ucsd.edu

*Sci. Adv.* **9**, eadi2960 (2023)
DOI: 10.1126/sciadv.adi2960

**The PDF file includes:**

Figs. S1 to S7
Tables S1 to S3
Legends for movies S1 to S4

**Other Supplementary Material for this manuscript includes the following:**

Movies S1 to S4

## Surface energies

The surface energies of one *low*-VEC and one *high*-VEC HEC composition are calculated by DFT using (i) 504 atoms divided in 6 atomic layers for {001} surfaces and (ii) 480 atoms divided in 6 layers for {110} and {111} surfaces. All structures have 1:1 metal/carbon ratio. Metal species are randomly arranged on the metal sublattice. Conjugate-gradient energy minimization is iterated to meet an accuracy of $10^{-5}$ eV/supercell while forces on each atom are smaller than $10^{-2}$ eV/Å.

Equilibrium structural parameters and bulk energy $E_{bulk}$ are determined by relaxing the cell shape and atomic positions in 3D-periodic DFT calculations. Thus, a vacuum region of 15 Å is added to separate slab replicas along the surface normal direction, and the atomic positions are relaxed. The surface energies are calculated as $E_{surf} = (E_{slab} - E_{bulk})/(2A)$, where 2A is the total surface area.

Consistent with DFT results of Ref. (*27*), obtained for binary systems, DFT calculations of the surface energies $E_{surf}$ of two representative HEC compositions (one *low*-VEC and one *high*-VEC) show that $E_{surf}^{\{001\}} < E_{surf}^{\{110\}} < E_{surf}^{\{111\}}$ (**Table S1**).

| HEC composition | Surface energies $E_{surf}$ (J/m$^2$) | | |
|---|---|---|---|
| | {001} | {110} | {111} |
| (Ti,Zr,Hf,Ta,W)C | 1.27 | 1.38 | 3.84 |
| (V,Nb,Ta,Mo,W)C | 0.84 | 1.23 | 3.12 |

**Table S1**. DFT surface energies of representative HEC compositions.

## Summary of AIMD mechanical properties

| HEC composition | $\sigma_T$ (GPa) | | $U_T$ (GPa) | | $\delta_f$ (%) | | Mechanism | |
|---|---|---|---|---|---|---|---|---|
| | [001] | [110] | [001] | [110] | [001] | [110] | [001] | [110] |
| (Ti,Zr,Hf,Nb,Ta)C | 26.1 | 38.2 | 1.40 | 2.14 | 10 | 12 | CL | CL |
| (Ti,Zr,Hf,Ta,W)C | 27.2 | 35.5 | 1.40 | 2.77 | 10 | 14 | CL | CL |
| (Ti,V,Nb,Ta,W)C | 26.7 | 31.8 | 2.39 | 3.35 | 12 | 18 | BF | BF |
| (V,Nb,Ta,Mo,W)C | 23.3 | 28.4 | 5.28 | 3.66 | 28 | 20 | TRIP | TRIP |
| (Ti,V,Cr,Mo,W)C | 25.4 | 28.9 | 4.23 | 6.16 | 22 | 34 | TRIP | TRIP |
| (V,Ta,Cr,Mo,W)C | 23.2 | 25.9 | 5.76 | 5.73 | 30 | 30 | TRIP | TRIP |

**Table S2**. Summary of theoretical tensile strengths $\sigma_T$, toughness $U_T$, and elongation at fracture $\delta_f$ calculated by AIMD simulations at room temperature for HEC uniaxially strained along the [001] and [110] crystallographic axes. The labels CL, TRIP, and BF, which indicate transformation and fracture mechanisms, stand for brittle "cleavage", "transformation-induced plasticity", and rapid "bond fraying", respectively.

## Chemistry measurements from EDS

| | Cr | Hf | Mo | Nb | Ta | Ti | V | W | Zr |
|---|---|---|---|---|---|---|---|---|---|
| (Ti,Zr,Hf,Nb,Ta)C | - | 20.5 | - | 19.4 | 20.5 | 20.0 | - | - | 19.6 |
| (Ti,Zr,Hf,Ta,W)C | - | 20.1 | - | - | 19.9 | 20.1 | - | 20.1 | 19.8 |
| (Ti,V,Nb,Ta,W)C | - | - | - | 19.2 | 20.7 | 20.7 | 19.5 | 19.9 | - |
| (V,Nb,Ta,Mo,W)C | - | - | 20.6 | 18.9 | 20.3 | - | 19.3 | 20.9 | - |
| (V,Ta,Cr,Mo,W)C | 20.0 | - | 20.5 | - | 19.5 | - | 19.5 | 20.5 | - |
| (V,Nb,Cr,Mo,W)C | 20.6 | - | 19.6 | 19.3 | - | - | 19.6 | 20.9 | - |

**Table S3**. Summary of sample chemistry as measured by EDS. The measured atomic percent of each cation in the HECs is in good agreement with the target 20 atom percent. Additionally, the targeted compositions match the measured chemistry.

## Cauchy pressures and G/B ratios vs VEC

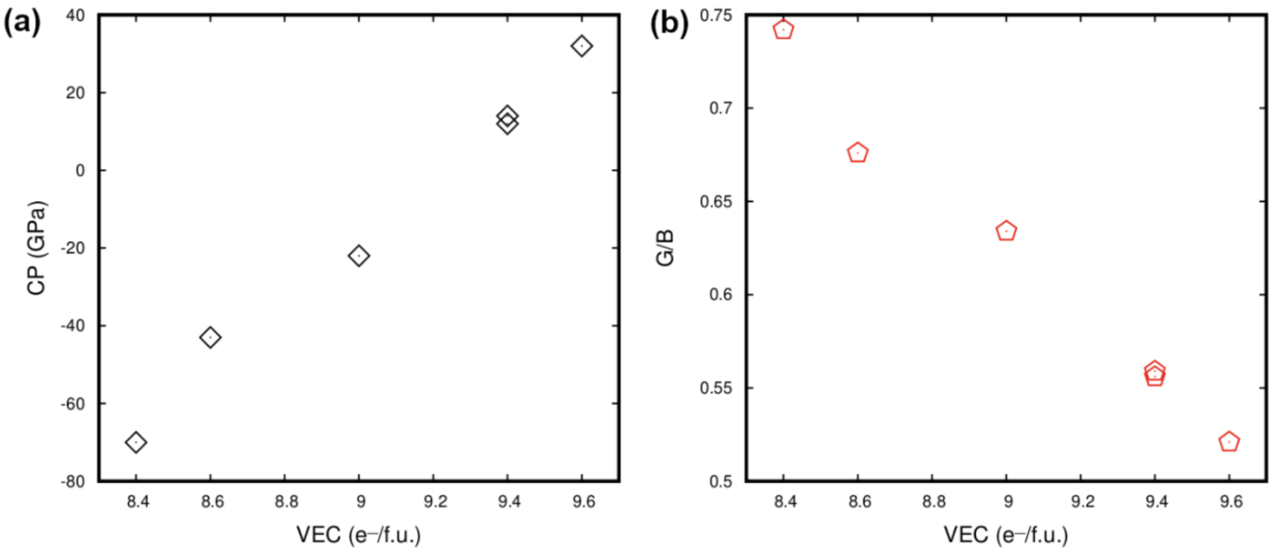


**Fig. S1**. Calculated Cauchy pressure and G/B ratios. **(a)** Cauchy pressure (CP) and **(b)** G/B ratio calculated by AIMD at 300 K and plotted as a function of the HEC alloy VEC. **Table 2** in the main text reports actual compositions.

**AIMD simulations of HECs uniaxially strained along [110]**

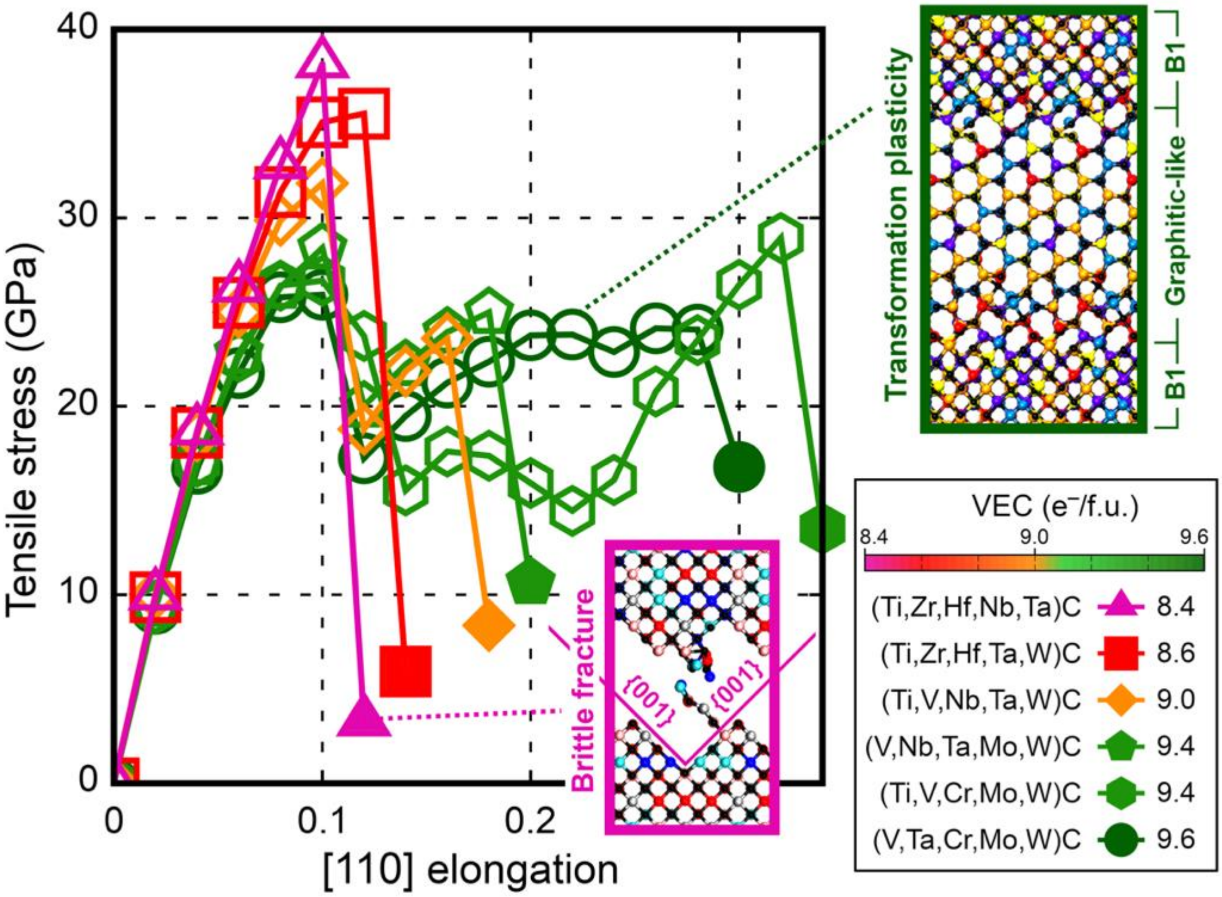


**Fig. S2**. **AIMD-calculated tensile-stress $\sigma^{[110]}$ vs. [110]-elongation of B1-structure HECs with different compositions and VEC.** The supercells used for [110] tensile-testing simulations are equivalent to those shown in figure 1b of Ref. (22). The filled symbols indicate fracture points. The insets illustrate AIMD snapshots of brittle fracture in (Ti,Zr,Hf,Nb,Ta)C (12% elongation) and strain-induced transformation plasticity in (V,Ta,Cr,Mo,W)C (22% elongation). Although the supercells are elongated parallel to the [110] crystallographic direction, (Ti,Zr,Hf,Nb,Ta)C cleaves along {001} surfaces, which have lowest formation energy (**Table S1**). Conversely, stress-activated B1→graphitic-like structural transformations significantly delay fracture in the high-VEC carbide (V,Ta,Cr,Mo,W)C. Chemical bonds have maximum length of 2.6 Å. Sphere color legend: black=C, silver=Ti, cyan=Zr, blue=Hf, ice-blue=V, pink=Nb, red=Ta, violet=Cr, yellow=Mo, orange=W.

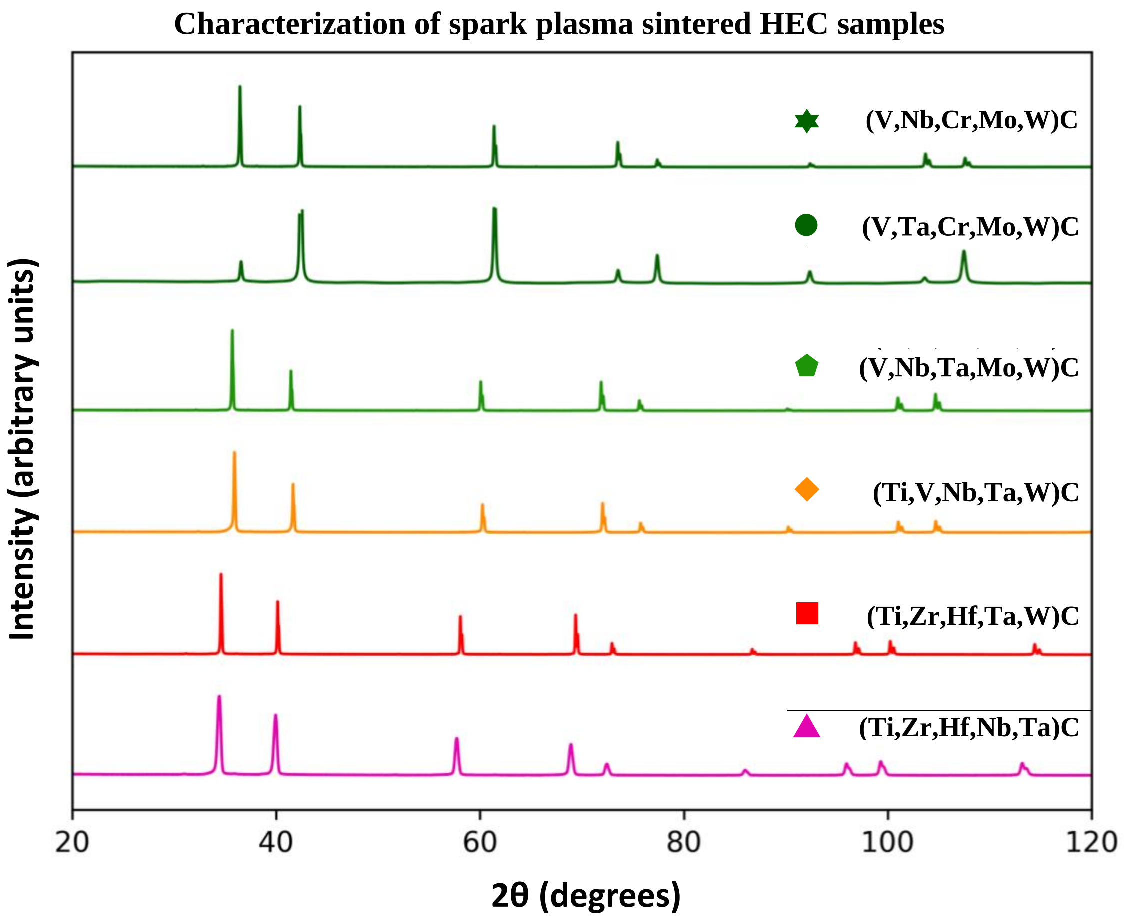


**Fig. S3**. **X-ray diffraction patterns for each of the bulk samples.** Only a rocksalt structure B1 phase is observed for each of the HECs.

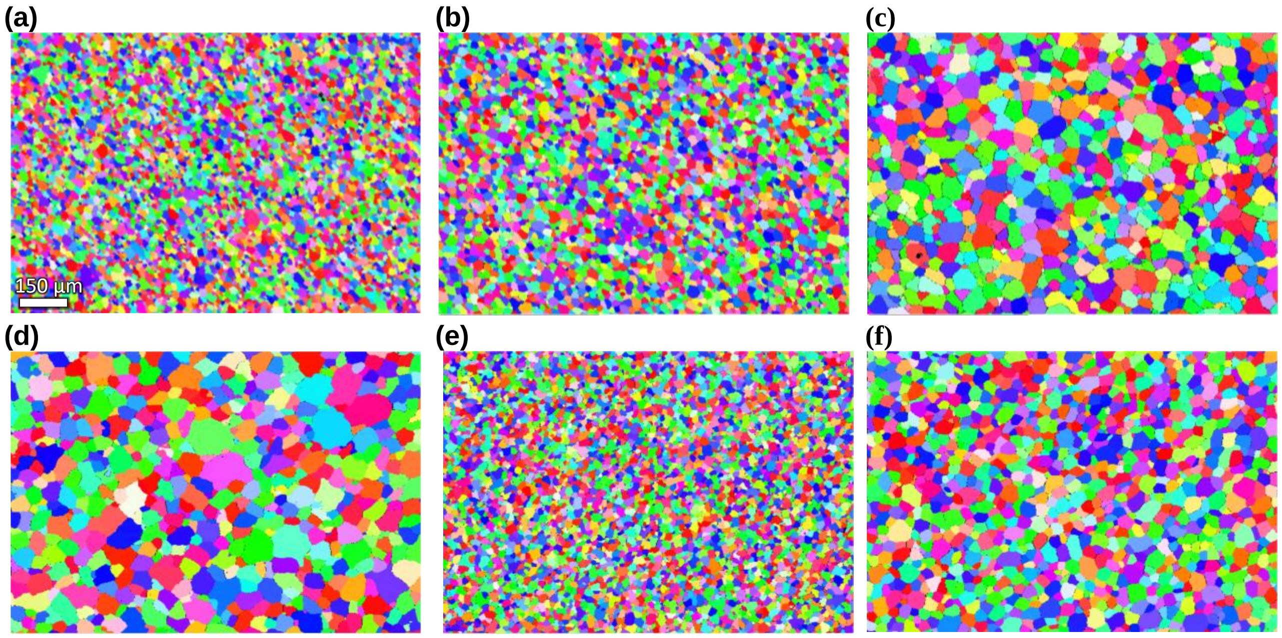


**Fig. S4**. **Electron backscatter diffraction orientation maps in the Z-direction (out-of-plane) for each of the six experimentally studied samples. (a)** (Ti,Zr,Hf,Nb,Ta)C, **(b)** (Ti,Zr,Hf,Ta,W)C, **(c)** (Ti,V,Nb,Ta,W)C, **(d)** (V,Nb,Ta,Mo,W)C, **(e)** (V,Ta,Cr,Mo,W)C, and **(f)** (V,Nb,Cr,Mo,W)C. The maps show highly equiaxed grains with average size ranging from ≈25-40 µm. Additionally, the low number of unindexed (black) pixels corroborates the high density measured via Archimedes' principle.

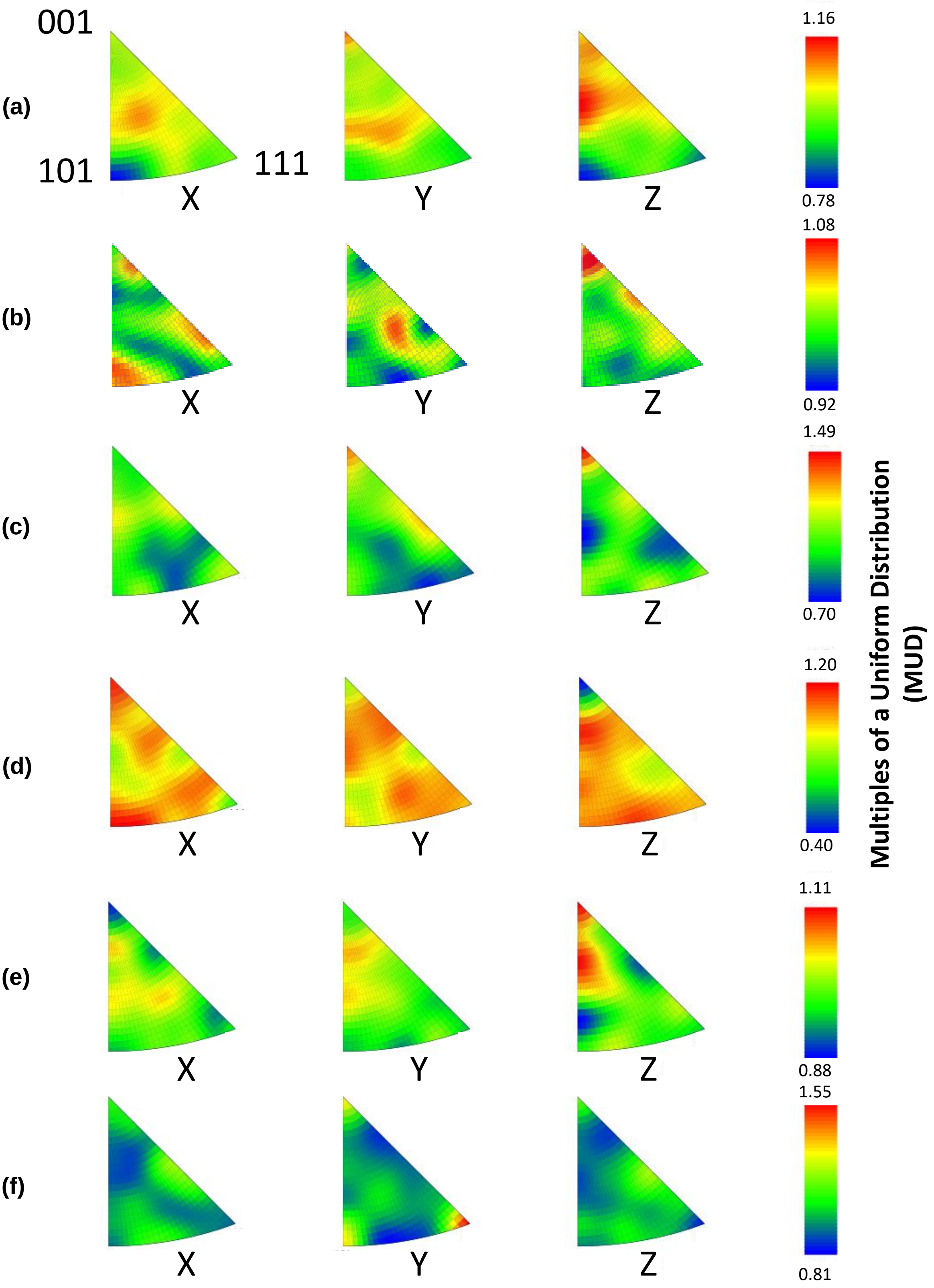


**Fig. S5**. **Texture analysis from the EBSD orientation maps.** For each of the samples **(a)** (Ti,Zr,Hf,Nb,Ta)C, **(b)** (Ti,Zr,Hf,Ta,W)C, **(c)** (Ti,V,Nb,Ta,W)C, **(d)** (V,Nb,Ta,Mo,W)C, **(e)** (V,Ta,Cr,Mo,W)C, and **(f)** (V,Nb,Cr,Mo,W)C, the maximum multiple of a uniform distribution (MUD) is 1.55. This denotes the samples have very low texture whereas medium texture starts at ≈5 MUD and highly textured is considered to start at ≈10 MUD.

## Fracture statistics during nanoindentation

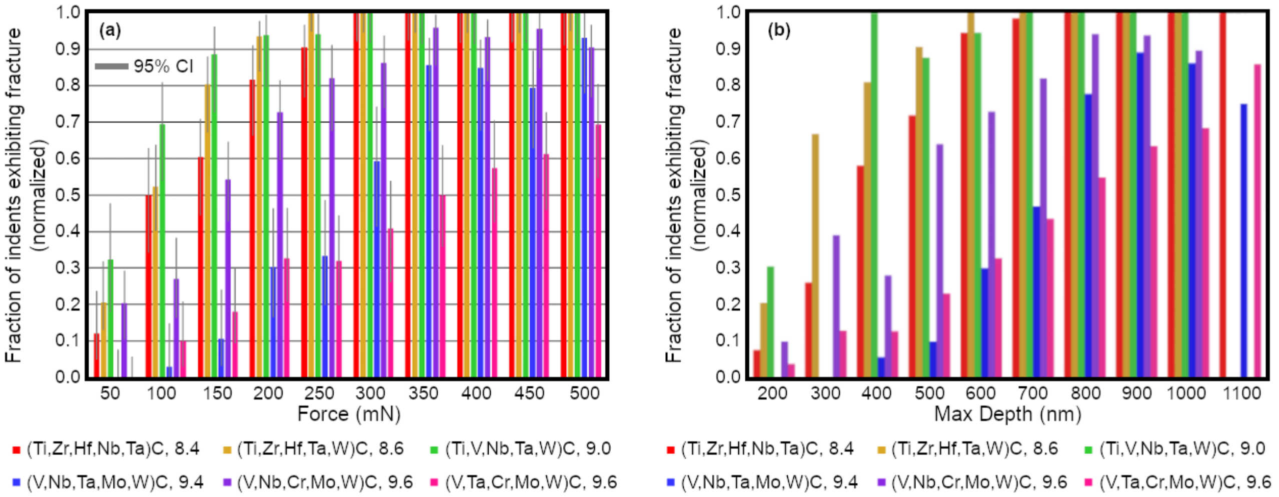


**Fig. S6**. **Statistics of indents exhibiting fracture.** The plots are at constant nanoindenter load (a) and binned penetration depth (b). Gray bars in (a) indicate confidence intervals (CI).

## Electron density maps of unstrained and strained HECs

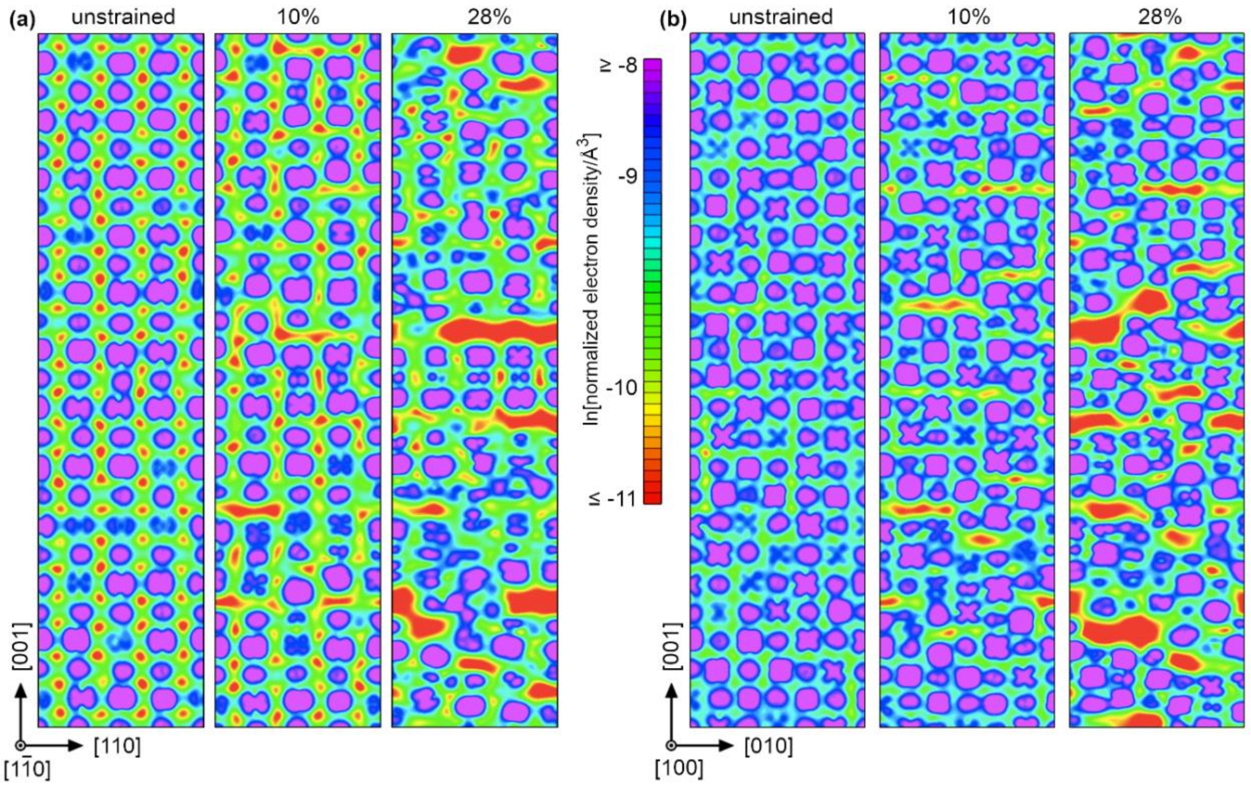


**Fig. S7**. Energy-resolved electron density of B1 (V,Ta,Cr,Mo,W)C calculated for vertical [001]-elongation of 0, 10, and 28% on **(a)** (1$\bar{1}$0) and **(b)** (100) lattice planes. The energy ranges used for electron-density calculations are [-2.9 eV to $E_F$] for the unstrained structure, [-2.3 eV to $E_F$] and [-2.0 eV to $E_F$] for 10 and 28% strained structures, respectively. These energy intervals are delimited by vertical orange-dotted and black-dashed lines in **Fig. 5a,b,c** of the main text. The electron densities are normalized by the total number of electrons (586, 529, and 502 $e^-$, for 0, 10, and 28% strain, respectively) included in the corresponding energy ranges. A logarithmic color scale is used to facilitate visualization of strain-induced changes in the electron arrangement. Off-scale electron densities ln(ρ') < -11 or ln(ρ') > -8 [where ρ' denotes a normalized density with units Å$^{-3}$] are colored in red and purple.

## Description of videos

AIMD videos of B1-structure HECs are generated using VMD (*71*). Videos of (Ti,Zr,Hf,Ta,W)C (VEC = 8.6 $e^{-}$/f.u.) and (V,Nb,Ta,Mo,W)C (VEC = 9.4 $e^{-}$/f.u.) subject to deformation can be found in (*26*). Here, atomic species are colored as: black=C, silver=Ti, cyan=Zr, blue=Hf, ice-blue=V, pink=Nb, red=Ta, violet=Cr, yellow=Mo, orange=W. The length of visible bonds is ≤ 2.6 Å. The resolution of the videos is ≈10–20 frames/ps. Each supercell contains 576 atoms (288 sites on each sublattice). The orientation of crystallographic directions relative to Cartesian axes is: $[1\bar{1}0]$//x, [110]//y, [001]//z. Supercells elongation parallel to [001] is increased stepwise with increments of 2%.

**Video captions**

**Video S1: AIMD simulation results of B1-structure (Ti,Zr,Hf,Nb,Ta)C [VEC = 8.4 $e^{-}$/ f.u.] subject to [001] elongation at room temperature.** The material cleaves before yielding.

**Video S2: AIMD simulation results of B1-structure (Ti,V,Nb,Ta,W)C [VEC = 9.0 $e^{-}$/f.u.] subject to [001] elongation at room temperature.** The material cleaves after very localized yielding.

**Video S3: AIMD simulation results of B1-structure (Ti,V,Cr,Mo,W)C [VEC = 9.4 $e^{-}$/f.u.] subject to [001] elongation at room temperature.** Transformation-induced plasticity at the yield point dissipates stress thus enhancing the material resistance to fracture.

**Video S4: AIMD simulation results of B1-structure (V,Ta,Cr,Mo,W)C [VEC = 9.6 $e^{-}$/ f.u.] subject to [001] elongation at room temperature.** Transformation-induced plasticity at the yield point dissipates stress thus enhancing the material resistance to fracture.
